# WNT7A/B assemble a GPR124-RECK-LRP5/6 coreceptor complex to activate β-catenin signaling in brain endothelial cells

**DOI:** 10.1016/j.jbc.2025.110682

**Published:** 2025-09-04

**Authors:** Robin Heiden, Laura Hannig, Calvin J. Kuo, Süleyman Ergün, Barbara M. Braunger, Mario Vallon

**Affiliations:** 1 Institute of Anatomy and Cell Biology, University of Würzburg, Würzburg, Germany; 2 Division of Hematology, Department of Medicine, Stanford University School of Medicine, Stanford, California, USA; 3 Istanbul Atlas University, Istanbul, Turkey

**Keywords:** WNT7A/B, WNT/β-catenin signaling, blood–brain barrier, brain endothelial cells, GPR124, RECK

## Abstract

WNT7A and WNT7B, secreted by neural cells, are essential regulators of developmental brain angiogenesis and blood–brain barrier integrity. In brain endothelial cells, WNT7 proteins activate β-catenin signaling through the ligand-specific receptor complex GPR124-RECK and classical WNT receptors of the FZD and LRP families. Previous studies suggested that WNT7 isoforms assemble a GPR124-RECK-FZD-LRP5/6 multireceptor complex for signaling. However, direct biochemical evidence for this complex and its signaling mechanisms remains elusive. Here, we investigated the formation and signaling mechanisms of WNT7 coreceptor complexes in brain endothelial cells using CRISPR/Cas9, biochemical analyses, and cell-based assays. Unexpectedly, cells with knockout of all FZD isoforms retained ∼25% of WNT7 responsiveness, whereas classical WNT3A signaling was completely abolished. Similarly, knockout of all *Dvl* paralogs, key mediators of FZD signaling, preserved ∼50% of WNT7 signaling activity but fully blocked WNT3A responses. In contrast, knockout of *Gpr124*, *Reck*, or *Lrp5/6* completely abrogated WNT7 signaling. Although both WNT7A and WNT3A triggered LRP6 phosphorylation, only WNT3A induced DVL2 phosphorylation. Biochemical analyses revealed WNT7-dependent recruitment of LRP5/6, but not FZD, to the GPR124-RECK heterodimer, forming a GPR124-RECK-WNT7-LRP5/6 complex. In GPR124-deficient cells, WNT7 proteins still assembled a RECK-WNT7-LRP5/6 core complex, yet this complex lacked signaling activity and LRP6 phosphorylation. Clustering of RECK-WNT7-LRP5/6 complexes with recombinant dimeric GPR124 ectodomain or a RECK antibody partially restored signaling, suggesting that GPR124 mediates formation of higher-order complexes. Our findings indicate that WNT7 signaling in brain endothelial cells is driven by distinct coreceptor complexes: a FZD-independent GPR124-RECK-LRP5/6 complex and FZD-dependent complexes that likely act in synergy.

Developmental central nervous system (CNS) angiogenesis, blood–brain barrier (BBB) formation, and BBB maintenance are critically regulated by Wingless-related integration site (WNT)/β-catenin signaling (1, 2, 3, 4, 5). WNT7A and WNT7B, secreted by neuroepithelial cells during development and by astrocytes postnatally, are the primary WNT isoforms that activate this signaling pathway in CNS endothelial cells (2, 3, 6). Unlike other WNTs, WNT7A/B require the receptors G protein–coupled receptor 124 (GPR124) and reversion-inducing cysteine-rich protein with Kazal motifs (RECK), alongside classical WNT receptors of the Frizzled (FZD) and low-density lipoprotein receptor–related protein (LRP) families for signaling (7, 8, 9, 10). Mice with single knockout of *Gpr124* or *Reck*, or double knockout of *Wnt7a/b* or *Lrp5/6*, exhibit remarkably similar CNS vascular phenotypes (3, 9, 11, 12), which can be rescued by stabilizing endothelial β-catenin (4, 7, 8). Interestingly, neither single nor double *Fzd* knockouts phenocopy these CNS vascular defects (13). However, *in vitro* overexpression experiments suggest that most FZD isoforms enhance WNT7 signaling in synergy with GPR124, RECK, and LRP5/6 (14).

GPR124, also known as ADGRA2 or TEM5, is essential for angiogenesis and vascular integrity in the developing forebrain and neural tube as well as for maintaining BBB integrity in ischemic stroke and gliobastoma (4, 11, 15). Despite its homology to G protein–coupled receptors, GPR124 does not activate G proteins (16). Moreover, GPR124 overexpression does not result in ligand-independent WNT/β-signaling (17), and its intracellular domain is dispensable for function *in vivo* (18), suggesting that intrinsic GPR124 signal transduction likely does not contribute to WNT7 signaling.

RECK, a glycosylphosphatidylinositol-anchored cell-surface protein, was initially identified as a tumor suppressor (19) and later as a metalloproteinase inhibitor (20). More recently, RECK has emerged as a key regulator of developmental CNS angiogenesis and BBB formation (7, 12, 14, 21). GPR124 and RECK form a constitutive complex in brain endothelial cells, and their purified recombinant ectodomains (ECDs) interact directly (7, 17). WNT7A/B bind to both free RECK and the RECK-GPR124 complex, with GPR124 not modulating the RECK-WNT7 interaction (17).

WNT/β-catenin signaling is typically initiated upon ligand binding to FZD and LRP5/6 coreceptors, leading to the formation of a FZD-WNT-LRP5/6 ternary complex (22). FZD subsequently recruits the intracellular scaffold protein Dishevelled (DVL), along with components of the β-catenin destruction complex. This recruitment triggers phosphorylation of both DVL and LRP5/6, followed by DVL-mediated clustering of WNT coreceptor complexes, also referred to as signalosome formation. WNT signalosomes inactivate the destruction complex, enabling intracellular stabilization and accumulation of β-catenin. In the nucleus, β-catenin associates with T cell factor (TCF) family transcription factors to induce WNT target gene expression.

WNT7A/B activate β-catenin signaling by engaging the ligand-specific receptor complex GPR124-RECK in addition to classical FZD and LRP5/6 receptors (7, 14, 17, 23). However, the precise mechanisms by which GPR124, RECK, FZD, and LRP5/6 cooperatively mediate WNT7 signaling remain unclear. Based on ectopic overexpression studies, it has been suggested that WNT7 proteins assemble all four receptors into a multiprotein complex for signaling (7, 21, 23, 24). We previously reported that RECK binding stabilizes WNT7 proteins in their active form, enhancing secondary FZD-WNT7 complex formation, suggesting sequential binding of WNT7 to its receptors (17). Despite these studies, direct biochemical evidence for such complexes in brain endothelial cells is lacking.

Here, we examine WNT7-induced coreceptor complex formation and signaling mechanisms in brain endothelial cells using CRISPR-mediated gene knockouts, cell-based assays, and biochemical analyses.

## Results

### WNT7 receptor requirements in brain endothelial cells

**Figure 1:**
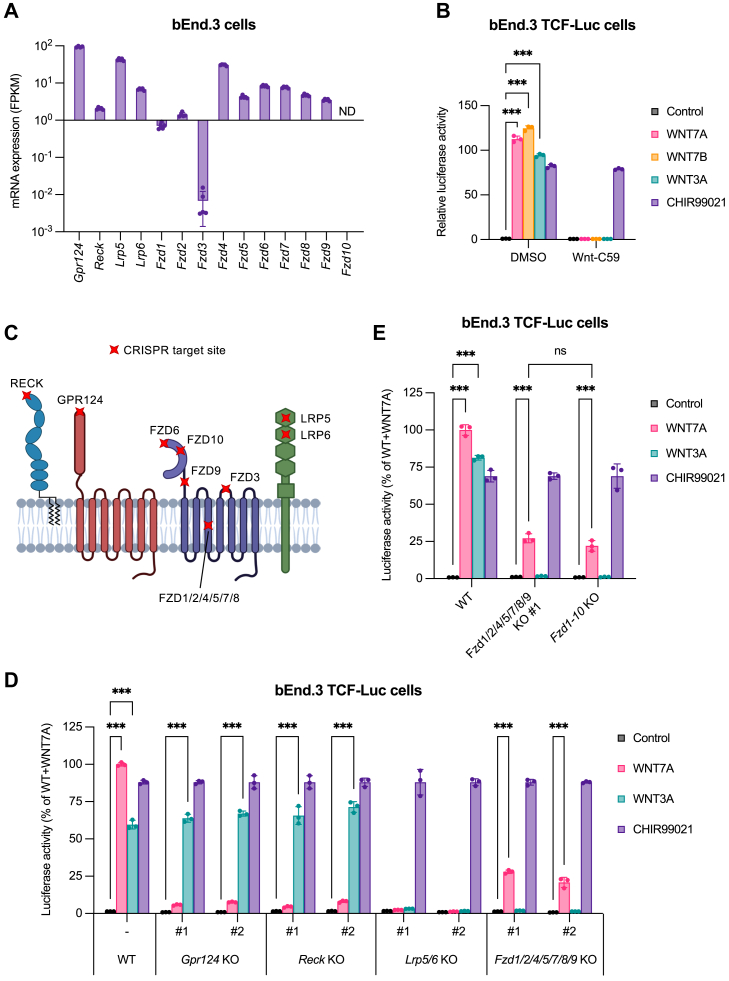
**WNT7 receptor requirements in brain endothelial cells**. *A*, RNA-seq analysis of WNT7 receptors in bEnd.3 cells (GEO #GSE268263, saline control). Bars show mean FPKM of biological replicates (n = 5) ± SD. *B*, WNT/β-catenin reporter assay using bEnd.3 TCF-Luc cells cocultured for 24 h with nonadherent parental HEK293 cells (control) or HEK293 cells expressing WNT7A, WNT7B, or WNT3A. Treatment with the GSK3 inhibitor CHIR99021 (10 μM) served as positive control. Cells were additionally treated with DMSO (vehicle control) or the porcupine inhibitor Wnt-C59 (1 μM). *Bars* represent luciferase activity as fold change over unstimulated, DMSO-treated cells (mean ± SD, n = 3, biological replicates). A two-way ANOVA with Tukey’s multiple comparisons test was used for statistical analysis. Only significant differences between control and WNT7A, WNT7B, or WNT3A stimulation within each condition are shown. Full statistical results are provided in Table S4. ∗∗∗*p* ≤ 0.001. *C*, schematic representation of WNT7 receptor domain structures and CRISPR target sites in bEnd.3 TCF-Luc cells. Created in BioRender. Heiden, R. (2025) https://BioRender.com/v58z453. *D* and *E*, WNT/β-catenin reporter assays using bEnd.3 TCF-Luc cells with indicated single or multiplex KO. Numbers denote independent KO clones. *Fzd1-10* KO cells were derived from *Fzd1/2/4/5/7/8/9* KO clone #1 with additional KO of *Fzd3, Fzd6,* and *Fzd10* (clone pool). WT or KO cells were cocultured for 24 h with nonadherent parental HEK293 cells (control) or HEK293 cells expressing WNT7A or WNT3A. Treatment with the GSK3 inhibitor CHIR99021 (10 μM) served as positive control. Bars represent luciferase activity normalized to CHIR99021 treatment and expressed as % of WNT7A-stimulated WT cells (mean ± SD, n = 3, biological replicates). A two-way ANOVA with Tukey’s multiple comparisons test was used for statistical analysis. Only significant differences between control and WNT7A or WNT3A stimulation within WT cells or KO clones are depicted. Full statistical results are provided in Table S4. ∗∗∗*p* ≤ 0.001; ns, not significant. FPKM, fragments per kilobase of transcript per million mapped reads; GEO, Gene Expression Omnibus; ND, nondetectable; CRISPR, clustered regularly interspaced short palindromic repeats. See also Figure S1 and Tables S1 and S2. WNT, Wingless-related integration site; FZD, Frizzled; TCF, T cell factor; DMSO, dimethyl sulfoxide.

To assess the individual contributions of the WNT7 receptors GPR124, RECK, FZD, and LRP5/6 to β-catenin signaling, we generated WNT/β-catenin reporter (TCF-Luc) and knockout sublines of the murine brain endothelial cell line bEnd.3. Analysis of RNA-seq data (GSE268263) revealed that bEnd.3 cells express all WNT7 receptors and most of their paralogs (Fig. 1*A*).

Unlike WNT3A, WNT7A and WNT7B rapidly form inactive aggregates after secretion, rendering commercially available recombinant WNT7A or WNT7-conditioned medium ineffective in WNT/β-catenin reporter assays (17). To stimulate bEnd.3 TCF-Luc cells with freshly secreted WNT, the cells were cocultured with HEK293 cells stably expressing WNT7A, WNT7B, or WNT3A, which led to robust reporter activation (Fig. 1*B*). Parental HEK293 cells served as control. Treatment of the cocultures with the porcupine inhibitor Wnt-C59 completely blocked reporter activation, indicating specificity of our approach.

Using the CRISPR/Cas9 system, we generated bEnd.3 TCF-Luc sublines with single knockout of *Gpr124* or *Reck*, double knockout of *Lrp5/6*, multiplex knockout of *Fzd1/2/4/5/7/8/9*, or knockout of all *Fzd* paralogs (*Fzd1-10*, Figs. 1*C* and S1, *A*–*O*, Tables S1 and S2). Surprisingly, both *Fzd1/2/4/5/7/8/9* and *Fzd1-10* knockout cells retained ∼25% of WNT7 signaling activity, whereas *Gpr124*, *Reck*, or *Lrp5/6* knockout completely abolished WNT7 responses (Figs. 1, *D* and *E* and S1, *P* and *Q*). In contrast, *Lrp5/6*, *Fzd1/2/4/5/7/8/9*, or *Fzd1-10* knockout fully abrogated classical WNT3A signaling, while *Gpr124* or *Reck* knockout had no effect. The effects on WNT signaling were reproducible across independent knockout clones (Figs. 1*D* and S1*P*).

### Ligand and receptor requirements for phosphorylation of LRP6 and DVL2 in brain endothelial cells

**Figure 2:**
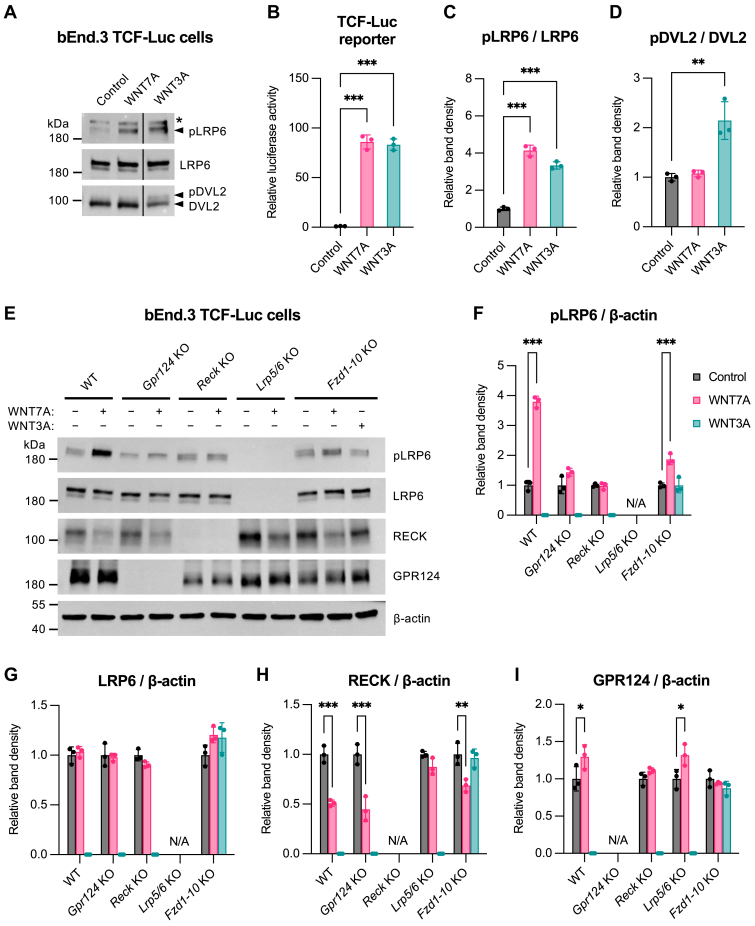
**Ligand and receptor requirements for phosphorylation of LRP6 and DVL2 in brain endothelial cells**. *A*–*D*, combined western blot analysis, band densitometry, and WNT/β-catenin reporter assay using bEnd.3 TCF-Luc cells cocultured for 24 h with nonadherent parental HEK293 cells (control) or HEK293 cells expressing WNT7A or WNT3A. Bars show mean (n = 3) ± SD from biological replicates as fold change over unstimulated cells. A one-way ANOVA with Tukey’s multiple comparisons test was used for statistical analysis. Only significant differences between control and WNT7A or WNT3A stimulation are shown. Full statistical results are provided in Table S4. ∗∗*p* ≤ 0.01, ∗∗∗*p* ≤ 0.001. *A*, representative western blot analysis. The pLRP6 antibody used detects LRP6 phosphorylated at Ser1490. The DVL2 antibody detects both non-phosphorylated and phosphorylated DVL2 (pDVL2), which exhibit differential migration in SDS-PAGE (band shift). The *asterisk* indicates a non-specific band. *B*, WNT/β-catenin reporter assay. *C*–*D*, western blot band density of pLRP6 and pDVL2 normalized to total LRP6 and total DVL2, respectively. *E*–*I*, western blot analysis and band densitometry using bEnd.3 TCF-Luc cells with indicated single or multiplex KO. Cells were cocultured for 24 h with nonadherent parental HEK293 cells (control) or HEK293 cells expressing WNT7A or WNT3A. Bars show mean band density (n = 3) ± SD from biological replicates as fold change over unstimulated cells. A two-way ANOVA with Tukey’s multiple comparisons test was used for statistical analysis. Only significant differences between control and WNT7A or WNT3A stimulation are shown. Full statistical results are provided in Table S4. ∗*p* ≤ 0.05, ∗∗*p* ≤ 0.01, ∗∗∗*p* ≤ 0.001. *E*, representative western blot analysis. The pLRP6 antibody used detects LRP6 phosphorylated at Ser1490. *F*–*I*, western blot band density of the indicated proteins normalized to β-actin. pLRP6, phospho-LRP6; pDVL2, phospho-DVL2. See also Figure S1 and Tables S1 and S2. WNT, Wingless-related integration site; LRP, lipoprotein receptor–related protein; TCF, T cell factor; Dvl, Dishevelled.

Phosphorylation of LRP5/6 and DVL is a hallmark of classical WNT/β-catenin signaling (22). To examine whether WNT7A elicits these phosphorylation events in bEnd.3 TCF-Luc cells, we performed western blot analyses side-by-side with a WNT reporter assay (Fig. 2, *A*–*D*). Stimulation of the cells with classical WNT3A served as positive control. Although both WNT7A and WNT3A triggered similar WNT reporter activation (Fig. 2*B*) and LRP6 phosphorylation (Fig. 2*C*), DVL2 phosphorylation was only induced by WNT3A (Fig. 2*D*).

Next, we investigated the contribution of the different WNT7 receptors to LRP6 phosphorylation (Fig. 2, *E* and *F*). Although *Gpr124* or *Reck* knockout completely blocked WNT7A-induced LRP6 phosphorylation (Fig. 2*F*), *Fzd1-10* knockout preserved ∼25% of WNT7A-induced, but not WNT3A-mediated, LRP6 phosphorylation. Quantification of LRP6, RECK, and GPR124 protein levels (band density, Fig. 2, *G*–*I*) revealed WNT7A-induced downregulation of RECK (Fig. 2*H*) and a slight tendency of GPR124 upregulation (Fig. 2*I*), whereas LRP6 remained largely unchanged (Fig. 2*G*). Notably, RECK downregulation was absent in *Lrp5/6* double knockout cells.

### Differential requirement for DVL in WNT7 versus WNT3A signaling

**Figure 3:**
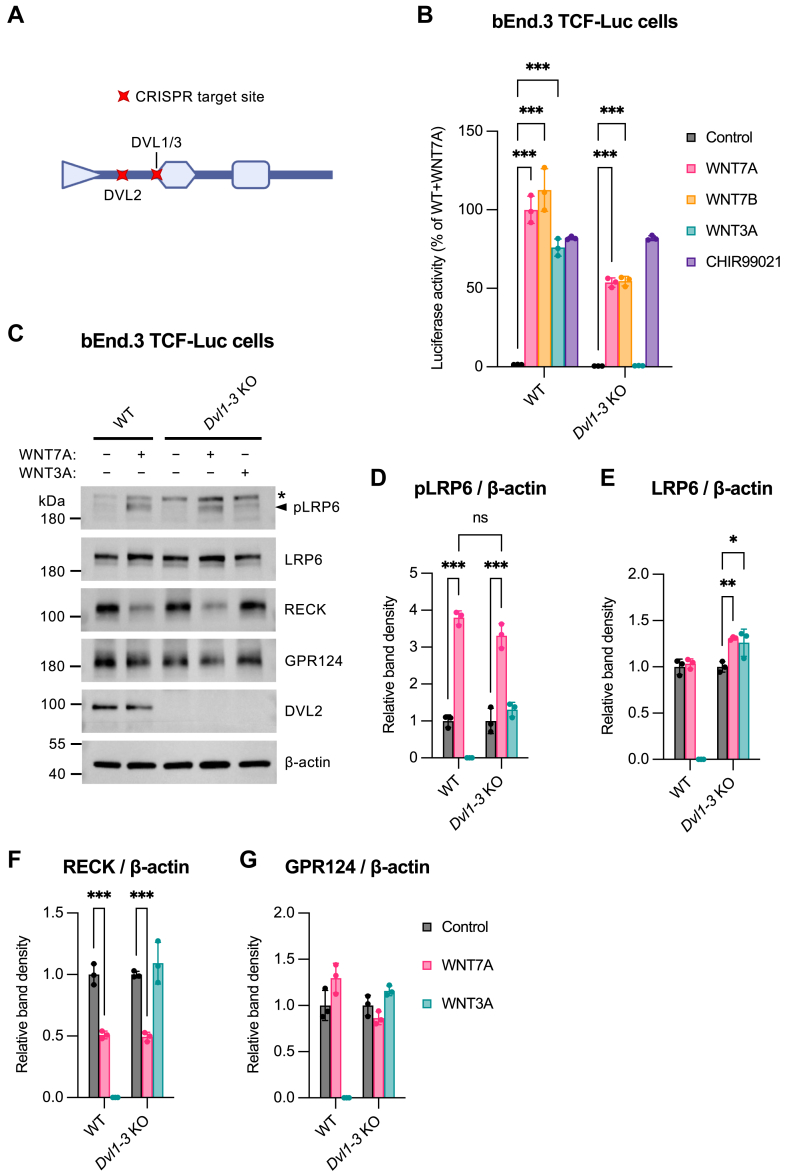
**Differential requirement for DVL in WNT7 *versus* WNT3A signaling**. *A*, schematic representation of DVL domain structure and CRISPR target sites in bEnd.3 TCF-Luc cells. Created in BioRender. Vallon, M. (2025) https://BioRender.com/236pjsb. *B*, WNT/β-catenin reporter assay using a *Dvl1-3* KO clone of bEnd.3 TCF-Luc cells. WT and KO cells were cocultured for 24 h with nonadherent parental HEK293 cells (control) or HEK293 cells expressing WNT7A, WNT7B, or WNT3A. Treatment with the GSK3 inhibitor CHIR99021 (10 μM) served as positive control. Bars represent luciferase activity normalized to CHIR99021 treatment and expressed as % of WNT7A-stimulated WT cells (mean ± SD, n = 3, biological replicates). A two-way ANOVA with Tukey’s multiple comparisons test was used for statistical analysis. Only significant differences between control and WNT7A, WNT7B, or WNT3A stimulation within WT cells or KO clone are shown. Full statistical results are provided in Table S4. ∗∗∗*p* ≤ 0.001. *C*–*G*, western blot analysis and band densitometry using a *Dvl1-3* KO clone of bEnd.3 TCF-Luc cells. WT and KO cells were cocultured for 24 h with nonadherent parental HEK293 cells (control) or HEK293 cells expressing WNT7A or WNT3A. Bars show mean band density (n = 3) ± SD from biological replicates as fold change over unstimulated cells. A two-way ANOVA with Tukey’s multiple comparisons test was used for statistical analysis. Only significant differences between control and WNT7A or WNT3A stimulation are shown. Full statistical results are provided in Table S4. ∗*p* ≤ 0.05, ∗∗*p* ≤ 0.01, ∗∗∗*p* ≤ 0.001; ns, not significant. *C*, representative western blot analysis. The pLRP6 antibody used detects LRP6 phosphorylated at Ser1490. The *asterisk* indicates a nonspecific band. *D*–*G*, western blot band density of the indicated proteins normalized to β-actin. CRISPR, clustered regularly interspaced short palindromic repeats; pLRP6, phospho-LRP6. See also Figure S2 and Tables S1 and S2. WNT, Wingless-related integration site; TCF, T cell factor; Dvl, Dishevelled

To further investigate the partial FZD-independence and absence of DVL2 phosphorylation in response to WNT7A, we generated a bEnd.3 TCF-Luc subline with knockout of all DVL isoforms (DVL1-3; Figs. 3*A* and S2). Interestingly, *Dvl1-3* knockout preserved ∼50% of WNT7 signaling activity, whereas WNT3A signaling was completely abolished (Fig. 3*B*). Strikingly, western blot analysis revealed similar levels of WNT7A-induced LRP6 phosphorylation in wild-type versus *Dvl1-3* knockout cells, while WNT3A-induced LRP6 activation was entirely blocked (Fig. 3, *C* and *D*). Quantification of other protein levels (band density) showed LRP6 upregulation upon WNT7A or WNT3A stimulation in knockout but not in wild-type cells (Fig. 3*E*). Moreover, WNT7A treatment downregulated RECK in both wild-type and knockout cells (Fig. 3*F*), whereas GPR124 protein levels remained unchanged under all conditions (Fig. 3*G*).

### WNT7A assembles a GPR124-RECK-LRP5/6 coreceptor complex in brain endothelial cells

**Figure 4:**
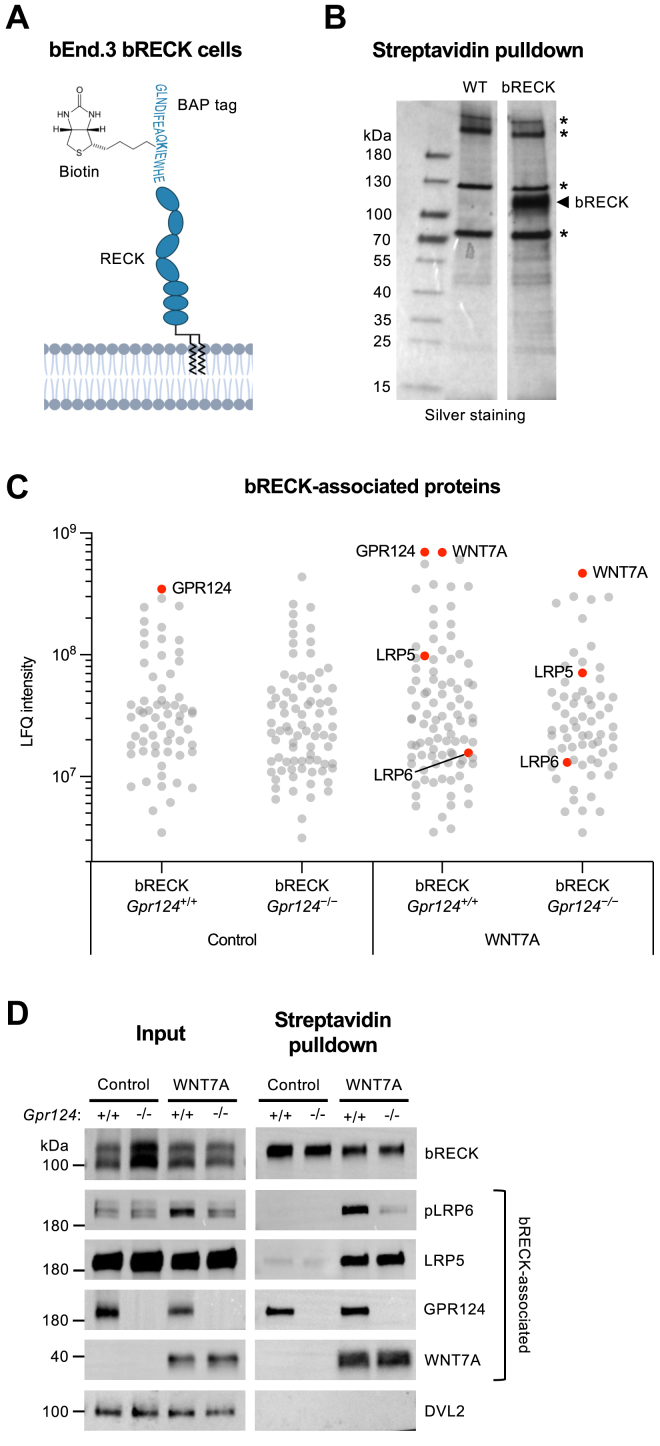
**WNT7A assembles a GPR124-RECK-LRP5/6 coreceptor complex in brain endothelial cells**. *A*, schematic illustrating biotinylated BAP-RECK (bRECK) expressed in bEnd.3 bRECK cells. Cells were generated by CRISPR-mediated KO of *Reck*, followed by stable transduction with a biotin acceptor peptide (BAP)-RECK fusion construct and the biotin ligase BirA. Created in BioRender. Heiden, R. (2025) https://BioRender.com/13oyhsp. *B*, SDS-PAGE analysis of streptavidin pull-downs from bEnd.3 WT or bRECK cells. Proteins were visualized by silver staining. *Asterisks* indicate endogenously biotinylated proteins. *C* and *D*, streptavidin pull-downs from bEnd.3 bRECK cells with or without *Gpr124* KO and with or without WNT7A stimulation were analyzed by (*C*) mass spectrometry and (*D*) western blotting. bEnd.3 bRECK cells were cocultured for 24 h with nonadherent parental HEK293 cells (control) or HEK293 WNT7A cells. Prior to cell lysis, protein-protein interactions were stabilized *in situ* by DSP cross-linking. Labeled *red dots* represent proteins that overlap with the GO term “canonical Wnt signaling pathway” (GO:0060070). The pLRP6 antibody used detects LRP6 phosphorylated at Ser1490. CRISPR, clustered regularly interspaced short palindromic repeats; LFQ, label-free quantification; DSP, dithiobis(succinimidylpropionate); GO, gene ontology; pLRP6, phospho-LRP6; WNT, Wingless-related integration site; GPR124, G protein–coupled receptor 124; RECK, reversion-inducing cysteine-rich protein with Kazal motifs; See also Figure S3 and Table S3.

We used a combined *in situ* chemical crosslinking and pull-down approach, to examine WNT7-induced coreceptor complex assembly in brain endothelial cells. bEnd.3 TCF-Luc cells were genetically engineered to express N-terminally biotinylated BAP-RECK (bRECK), which maintained functional activity in WNT7 signaling (Figs. 4*A* and S3, *A* and *B*). Streptavidin pull-down of bRECK from bEnd.3 bRECK cells resulted in partial purification, with coisolation of endogenously biotinylated proteins (Fig. 4*B*). To assess GPR124-dependent recruitment of proteins to bRECK, we generated a bEnd.3 bRECK *Gpr124*^*−/−*^ subline (Fig. S3, *C* and *D*).

bEnd.3 bRECK cells, with or without *Gpr124* knockout, were stimulated with WNT7A or left unstimulated. Protein-protein interactions were stabilized *in situ* using the thiol-cleavable chemical crosslinker dithiobis(succinimidylpropionate) (DSP), followed by streptavidin pull-down of bRECK and associated proteins. A mock streptavidin pull-down from bEnd.3 *Reck*^*−/−*^ cells served as a negative control. bRECK-associated proteins were eluted from the beads by cleaving the DSP crosslinks with DTT. The eluted proteins were identified and quantified by mass spectrometry (Fig. 4*C*, Table S3). Only proteins absent from the negative control were considered specific bRECK interactors.

Across all four conditions, the mass spectrometry analysis identified 134 bRECK-associated proteins. Four of these overlapped with the gene ontology (GO) term “canonical Wnt signaling pathway” (GO:0060070): GPR124, WNT7A, LRP5, and LRP6 (Fig. 4*C*). Notably, none of the ten FZD isoforms was detected. As expected, in stimulated *Gpr124*^*+/+*^ cells, GPR124 and WNT7A were the most abundant bRECK-associated proteins based on label-free quantification (LFQ) intensities. Intriguingly, the WNT coreceptors LRP5 and LRP6 were recruited to bRECK upon WNT7A stimulation, and this association occurred independently of GPR124. Consequently, in *Gpr124*^*+/+*^ cells, WNT7A stimulation induced assembly of a GPR124-bRECK-WNT7A-LRP5/6 complex, whereas in *Gpr124*^*−/−*^ cells, WNT7A stimulation resulted in formation of a bRECK-WNT7A-LRP5/6 core complex. The known RECK binding partner and inhibitory target ADAM10 (25) as well as its homolog ADAM15 were identified across all conditions, confirming specificity of our approach (Table S3). Unbiased GO molecular function analysis of bRECK-associated proteins revealed enrichment in GO terms related to cell-cell and cell-matrix adhesion, growth factor binding, transmembrane receptor protein kinase activity, Wnt receptor activity, and Wnt-protein binding, aligning with the known functions of RECK (26) (Fig. S3*E*, Table S3).

Western blot analysis of the streptavidin pull-downs confirmed WNT7A-dependent recruitment of LRP5 to bRECK, independently of GPR124 (Fig. 4*D*). Phospho-LRP6 western blotting further revealed enhanced LRP5/6 activation in the GPR124-bRECK-WNT7A-LRP5/6 complex compared to the bRECK-WNT7A-LRP5/6 core complex, suggesting that GPR124 modulates LRP5/6 activation rather than recruitment.

### Clustering of RECK-WNT7-LRP5/6 complexes partially compensates for GPR124 deficiency

Although HEK293 cells express all WNT7 receptors on the mRNA level (Fig. S4*A*), expression of GPR124 on the protein level is negligible and undetectable by western blotting (17). Hence, HEK293 cells are not intrinsically responsive to WNT7 stimulation and require GPR124 overexpression (8, 14). We previously reported that recombinant soluble GPR124 ECD can substitute for transfected full-length GPR124 in mediating WNT7 signaling in HEK293 cells (17). Building on these findings, we show here that the GPR124 ECD supports WNT7 signaling only when present as a dimer.

**Figure 5:**
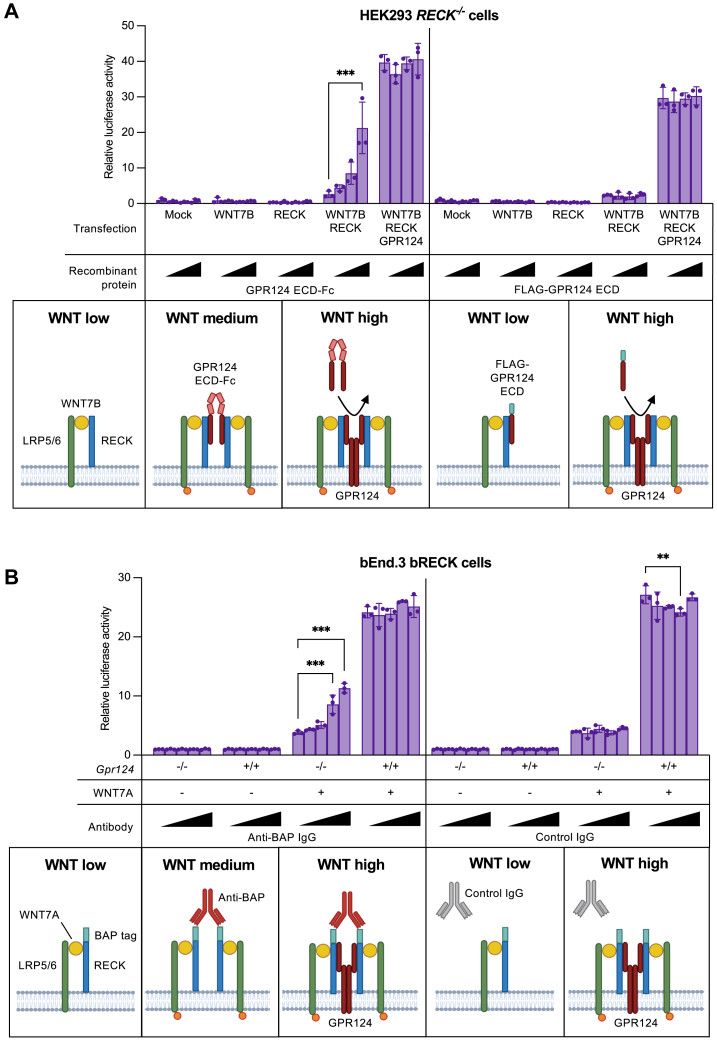
**Clustering of RECK-WNT7-LRP5/6 complexes partially rescues GPR124 deficiency.***A*, *Top*: WNT/β-catenin reporter gene assay using HEK293 *RECK*^*−/−*^ cells cotransfected with empty vector (mock) or the indicated expression vectors and a TCF-Luc reporter. Twenty-four hours after transfection, cells were treated with the indicated recombinant protein at 0, 1, 10, or 100 nM (wedges) for 24 h. Bars represent luciferase activity as fold change over mock-transfected cells (mean ± SD, n = 3, biological replicates). A two-way ANOVA with Tukey’s multiple comparisons test was used for statistical analysis. Only significant differences between 0 and 1, 10, or 100 nM protein within each condition are shown. Full statistical results are provided in Table S4. ∗∗∗*p* ≤ 0.001. *Bottom*: Schematics depicting clustering-induced activation of RECK-WNT7B-LRP5/6 complexes. Dimerization of full-length GPR124 is hypothetical. Created in BioRender. Heiden, R. (2025) https://BioRender.com/bvq7ldl. *B*, *Top*: WNT/β-catenin reporter gene assay using bEnd.3 bRECK cells with or without *Gpr124* KO, stably transfected with a TCF-Luc reporter. Cells were cocultured with nonadherent parental HEK293 cells or HEK293 WNT7A cells and treated for 12 h with the indicated antibody at 0, 0.01, 0.1, 1, or 10 μg/ml (wedges). *Bars* represent luciferase activity as fold change over unstimulated cells at the same antibody concentration (mean ± SD, n = 3, biological replicates). A two-way ANOVA with Tukey’s multiple comparisons test was used for statistical analysis. Only significant differences between 0 and 0.01, 0.1, 1, or 10 μg/ml antibody within each condition are shown. Full statistical results are provided in Table S4. ∗∗*p* ≤ 0.01, ∗∗∗*p* ≤ 0.001. *Bottom*: schematics depicting clustering-induced activation of bRECK-WNT7A-LRP5/6 complexes. Dimerization of full-length GPR124 is hypothetical. Created in BioRender. Heiden, R. (2025) https://BioRender.com/5jhi3m7. ECD, ectodomain; Fc, IgG Fc fragment; SD, standard deviation; BAP, biotin acceptor peptide; bRECK, biotinylated BAP-RECK. See also Figure S4. WNT, Wingless-related integration site; GPR124, G protein–coupled receptor 124; RECK, reversion-inducing cysteine-rich protein with Kazal motifs; TCF, T cell factor.

Cotransfection of HEK293 *RECK*^*−/−*^ cells with GPR124, RECK, and WNT7B resulted in robust WNT/β-catenin reporter activation (Fig. 5*A*). In contrast, cotransfection with RECK and WNT7B alone only mediated weak reporter activation. However, supplementing the culture medium of these cells with dimeric GPR124 ECD-Fc protein dose dependently enhanced reporter activation, whereas monomeric FLAG-GPR124 ECD had no effect (Figs. 5*A* and S4, *B* and *C*). These findings suggest that binding of dimeric GPR124 ECD-Fc to RECK-WNT7B-LRP5/6 complexes, driven by the known GPR124-RECK interaction (17), results in dimerization or higher-order clustering of RECK-WNT7B-LRP5/6 complexes to enable signaling (Fig. 5*A*, bottom panel). Notably, reporter activation in GPR124/RECK/WNT7B cotransfected cells remained unaffected by treatment with recombinant GPR124 ECD proteins, indicating that overexpressed full-length GPR124 already engages all RECK-WNT7B-LRP5/6 complexes and is not displaced by the recombinant proteins at concentrations up to 100 nM.

Based on our results in HEK293 cells, we hypothesized that clustering the signaling-deficient bRECK-WNT7A-LRP5/6 complex in bEnd.3 bRECK *Gpr124*^*−/−*^ cells might restore signaling. Indeed, a bivalent antibody targeting the biotin acceptor peptide (BAP) of bRECK dose dependently enhanced WNT7A-induced reporter activation (Fig. 5*B*). This indicates that anti-BAP IgG treatment induces dimerization or higher-order clustering of bRECK-WNT7A-LRP5/6 complexes, promoting WNT/β-catenin signaling. In contrast, anti-BAP IgG treatment had no effect on unstimulated bEnd.3 bRECK *Gpr124*^*−/−*^ cells. WNT7A stimulation robustly activated the reporter in bEnd.3 bRECK *Gpr124*^*+/+*^ cells and was unaffected by anti-BAP IgG, indicating that endogenous GPR124 already mediates maximum signaling. These findings suggest that the role of GPR124 in WNT7 signaling is dimerization or higher-order clustering of RECK-WNT7-LRP5/6 complexes, leading to FZD-independent activation of LRP5/6, as previously described (27, 28).

## Discussion

WNT/β-catenin signaling in brain endothelial cells is critical for developmental CNS angiogenesis and BBB integrity, with WNT7A and WNT7B serving as key ligands in these processes. Genetic studies in mice and complementary *in vitro* studies have established that WNT7 signaling requires the receptors GPR124, RECK, FZD, and LRP5/6 (7, 8, 9, 14, 23). However, the precise mechanisms by which these receptors mediate WNT7 signaling remain poorly understood. Here, we demonstrated that WNT7 proteins assemble a GPR124-RECK-LRP5/6 coreceptor complex, which acts partially independent of the FZD-DVL axis. Our results indicate that WNT7 signaling operates through at least two synergistic pathways: a core FZD-independent pathway driven by the GPR124-RECK-LRP5/6 complex and a FZD-DVL-dependent arm that amplifies the response. These findings challenge previous models proposing GPR124-RECK-FZD-LRP5/6 multireceptor complex formation (7, 23, 24) or sequential ligand binding to RECK and FZD (17).

Knockout of all *Fzd* paralogs in brain endothelial cells preserved ∼25% of WNT7 signaling activity, indicating that FZDs are important but nonessential for WNT7 signaling. This contrasts with prior findings in GPR124/RECK/WNT7A cotransfected HEK293 cells, where *FZD1-10* knockout completely abolished WNT reporter activation (23). The discrepancy may arise from differences in WNT reporter sensitivity, cell type (brain endothelial *versus* nonendothelial), or receptor expression levels (endogenous *versus* overexpressed). Similarly, knockout of all DVL isoforms preserved ∼50% of WNT7 responsiveness but fully abrogated classical WNT3A responses, confirming partial independence from the FZD-DVL axis. The differential dependence of WNT7 signaling on FZD and DVL (75% *versus* 50%) may reflect DVL-independent FZD functions or upregulation of FZD and/or LRP5/6 upon DVL knockout, as previously reported (29). Unlike WNT3A, WNT7A did not induce DVL2 phosphorylation, possibly because DVL2 phosphorylation is not required, occurs at low levels, is transient, or is not detectable by standard methods (band shift).

Knockout of *Gpr124*, *Reck*, or *Lrp5/6* in brain endothelial cells abolished WNT7 signaling completely, identifying them as core components of the WNT7 signaling machinery. Consistently, biochemical analyses revealed WNT7-dependent recruitment of LRP5/6, but neither FZD nor DVL isoforms, to the constitutive GPR124-RECK complex. The lack of FZD recruitment to the GPR124-RECK-WNT7 complex aligns with our previous findings that RECK-WNT7 and FZD-WNT7 interactions are mutually exclusive and that the soluble GPR124-RECK-WNT7 complex does not interact with soluble FZD ECDs (17). This indicates that FZDs might act within distinct classical FZD-LRP5/6 complexes synergizing with the GPR124-RECK-LRP5/6 complex (Fig. S5). However, the absence of FZD recruitment to the GPR124-RECK-WNT7 contrasts with prior studies demonstrating binding of a soluble RECK fragment (CC1-5) to GPR124/FZD/WNT7A-transfected HEK293T cells (7, 21) or constitutive GPR124-FZD colocalization in zebrafish blastulae overexpressing these receptors (24). The discrepancies may arise from differences in the cell systems (brain endothelial cells *versus* HEK293T or blastula cells) or receptor expression levels (endogenous *versus* overexpressed). Alternatively, FZDs may have evaded detection in our system due to transient interactions, incomplete recruitment, or technical limitations and function as a nonessential component of the previously proposed GPR124-RECK-FZD-LRP5/6 multireceptor complex.

We previously established that WNT7 isoforms directly bind to RECK and LRP5/6 but not to GPR124 (17). Accordingly, LRP5/6 is likely recruited to the GPR124-RECK complex by simultaneous binding of WNT7 to RECK and LRP5/6, forming a GPR124-RECK-WNT7-LRP5/6 quaternary complex. In *Gpr124* knockout cells, WNT7 stimulation still induced RECK-WNT7-LRP5/6 ternary complex formation, yet this complex lacked signaling activity and LRP6 phosphorylation. This indicates that GPR124 is essential for LRP5/6 activation but not recruitment. Interestingly, WNT7A stimulation resulted in selective downregulation of RECK protein, which depended on LRP5/6 but not on GPR124, FZD, or DVL. Since downregulation occurred in both transcriptionally responsive (WT) and nonresponsive (KO) cells, it unlikely represents negative feedback on the transcriptional level. Instead, the GPR124-RECK-WNT7A-LRP5/6 complex may undergo internalization and degradation as a negative feedback mechanism. Selective downregulation of RECK might be a result of targeted degradation or shedding, or GPR124 and LRP5/6 codegradation might be less apparent due to their higher abundance.

Notably, soluble dimeric (Fc fusion) but not monomeric (FLAG-tagged) GPR124 ECD enhanced WNT reporter activation in RECK/WNT7B-transfected HEK293 cells, likely by mediating dimerization or higher-order clustering of the signaling-deficient RECK-WNT7B-LRP5/6 complex. Similarly, antibody-mediated crosslinking of RECK-WNT7A-LRP5/6 complexes in *Gpr124* knockout brain endothelial cells partially rescued WNT reporter activation. These findings align with prior reports demonstrating that LRP5/6 can be activated FZD-independently upon dimerization or oligomerization (27, 28) and suggest that GPR124 facilitates this process within the GPR124-RECK-WNT7-LRP5/6 complex. GPR124-mediated clustering may explain its essential role in WNT7 signaling, potentially compensating for DVL’s role in WNT signalosome formation (22). Although dimeric GPR124 ECD-Fc and anti-BAP IgG both bind (BAP-)RECK bivalently, it is unclear whether this results in RECK dimerization or higher-order clustering. It has been reported that RECK forms homodimers intrinsically (30), which would result in higher-order oligomerization upon additional extrinsic dimerization. The inability of artificial clustering of RECK-WNT7-LRP5/6 complexes to fully restore β-catenin signaling may reflect native GPR124 functions, such as optimal spatial organization or oligomerization of the complex. Alternatively, the partial rescue could be a result of lower affinity of GPR124 ECD-Fc or the BAP antibody compared to transmembrane GPR124. Further studies are needed to confirm whether full-length GPR124 forms constitutive or ligand-induced dimers/oligomers as previously demonstrated for other G protein–coupled receptors (31, 32).

Our findings advance the understanding of the molecular mechanisms governing CNS vascular development and BBB maintenance, revealing that WNT7 signaling in brain endothelial cells is partially driven by a FZD-independent GPR124-RECK-LRP5/6 coreceptor complex. Future work should also explore whether this FZD-independent mechanism extends to other WNT7-responsive cell types or developmental contexts beyond brain endothelial cells.

## Experimental procedures

### Cell culture

The murine brain endothelial cell line bEnd.3 was obtained from the American Type Culture Collection (#CRL-2299) (33). The murine retroviral packaging cell line GP+E-86 (34) was kindly provided by Tatjana Hirsch (Helmholtz Centre for Infection Research, Braunschweig). HEK293 and HEK293T cells were a gift from Markus Sauer (University of Würzburg). All cell lines were maintained in Dulbecco's modified Eagle's medium (Thermo Fisher Scientific, 41966052) supplemented with 10% fetal calf serum (Biowest, S00H81000C) and 10 μg/ml gentamicin (Merck, 157500601). Cells were cultured at 37 °C in a humidified atmosphere with 5% CO_2_. All experiments were performed with cells free from mycoplasma contamination, which was routinely tested by PCR.

### Generation of bEnd.3 TCF-Luc cells

Subsequently, 1 × 10^5^ bEnd.3 cells were seeded into one well of a six-well plate and transfected with 2 μg of the WNT/β-catenin reporter construct pGL4.49[luc2P/TCF-LEF/Hygro] (TCF-Luc, Promega, E461A) using the jetOPTIMUS transfection reagent (Polyplus, 101000025). Forty-eight hours after transfection, cells were incubated with 500 μg/ml hygromycin (Thermo Fisher Scientific, J60681.03) for 7 days to eliminate nontransfected and transiently transfected cells. Single-cell clones of stably transfected cells were isolated by limiting dilution cloning and analyzed in a WNT/β-catenin reporter assay. The clone with the highest WNT responsivity was used for downstream experiments.

### Generation of HEK293 cells stably expressing WNT7A, WNT7B, or WNT3A

Subsequently, 4 × 10^5^ HEK293 cells per well were seeded into a six-well plate and transfected with 2 μg pcDNA-WNT3A, 2 μg pcDNA-WNT7A, or 2 μg pcDNA-WNT7B (gifts from Marian Waterman; Addgene #35908, #35914, and #35915) (35) using the jetOPTIMUS transfection reagent (Polyplus, 101000025). Twenty-four hours post transfection, cells were incubated with 600 μg/ml geneticin (Capricorn Scientific, G418-Q) for ∼14 days to select for stably transfected cells. Cells were continuously cultured in medium supplemented with 600 μg/ml geneticin to maintain selection pressure.

### WNT/β-catenin reporter gene assay

Briefly, 1 × 10^4^ bEnd.3 TCF-Luc cells or sublines were seeded per well into 96-well plates and incubated overnight until confluent. Reporter cells were overlaid with 2 × 10^4^ parental HEK293 cells (control) or HEK293 cells expressing WNT7A, WNT7B, or WNT3A and cocultured for 12 to 24 h. Where indicated, cocultures were treated with the pharmacological β-catenin activator CHIR99021 (10 μM, Biomol, Cay13122-25) (36) or the Porcupine inhibitor Wnt-C59 (1 μM, MedChemExpress, HY-15659). A luciferase assay (Promega, E1501) was performed according to the manufacturer's instructions. Bioluminescence (luciferase activity) was measured using the FLUOstar Omega plate reader (BMG-LABTECH).

### CRISPR/Cas9-mediated gene knockout

Guide RNAs (gRNAs) targeting the coding sequences of genes of interest were designed using the CRISPRdirect online tool (https://crispr.dbcls.jp/) (37) (Table S1). *Fzd1/2/4/5/7/8* were knocked out using a single gRNA targeting the highly conserved transmembrane helix 3 (TM3). Similarly, *Dvl1* and *Dvl3* were knocked out using a single gRNA targeting a highly conserved region in these paralogs. gRNAs were synthesized as sense and antisense ssODN (Thermo Fisher Scientific) and cloned into the vector pSpCas9(BB)-2A-Puro (PX459) V2.0 (a gift from Feng Zhang, Addgene #62988) (38), which coexpresses the gRNA and Cas9. bEnd.3 TCF-Luc cells were seeded into six-well plates (1 x 10^5^ cells per well) and transfected with 2 μg of the corresponding CRISPR construct using the jetOPTIMUS transfection reagent (Polyplus, 101000025). For double (*Lrp5/6*) or multiplex knockouts (*Fzd1/2/4/5/7/8/9*, *Dvl1-3*), the respective CRISPR plasmids were cotransfected at a 1:1 ratio. Forty-eight hours after transfection, cells were treated with 4 μg/ml puromycin (Thermo Fisher Scientific, A1113803) for 48 h to select for transiently transfected cells. Single-cell clones were isolated by limiting dilution cloning. To generate *Fzd1-10* KO cells, a stepwise transfection approach was used: *Fzd1/2/4/5/7/8/9* KO clone one was cotransfected with *Fzd3* and *Fzd6* CRISPR constructs, followed by puromycin selection. The resulting clone pool was expanded for 7 days, transfected with the *Fzd10* CRISPR plasmid, and again subjected to puromycin selection. This clone pool was used for downstream experiments. The targeted genomic regions were amplified by PCR (Table S2) and sequenced using the Sanger method (Microsynth Seqlab). Mixed sequencing chromatograms from single-cell clones were deconvoluted using the CRISP-ID software (39) or the DECODR online tool (https://decodr.org/) (40). Only clones with indels in all alleles (up to four) were considered knockouts and used for downstream experiments. For the *Fzd1-10* KO clone pool, mixed sequencing chromatograms from the *Fzd3*, *Fzd6*, and *Fzd10* target sites were deconvoluted using the TIDE online tool (https://apps.datacurators.nl/tide/) (41). Knockout induction was further validated by western blotting.

### Western blot analysis and quantification

Indicated bEnd.3 sublines were seeded into six-well plates (2 × 10^5^ cells per well) or T175 flasks (3 × 10^6^ cells per flask) and incubated until confluent. Cell monolayers were cocultured with nonadherent parental HEK293 cells or HEK293 cells expressing WNT7A, WNT7B, or WNT3A for 12 to 24 h (4 × 10^5^ cells per six-well, 2 × 10^7^ cells per T175 flask). Cells were washed twice with ice-cold PBS, which completely removed the nonadherent HEK293 cells. bEnd.3 cells were scraped into ice-cold RIPA buffer (25 mM Tris–HCl, pH 7.4; 150 mM NaCl; 1% Triton X-100; 0.5% sodium deoxycholate; and 0.1% SDS) supplemented with cOmplete ULTRA protease inhibitor (Merck, 5892970001) and PhosSTOP phosphatase inhibitor (Merck, 4906845001) cocktails. Cell lysates were incubated on ice for 30 min, vortexing occasionally. Insoluble material was removed by high-speed centrifugation, samples were taken, and lysates were stored at −80 °C. Samples (20 μg protein each) were supplemented with reducing (50 mM DTT) Laemmli sample buffer, incubated at 70 °C for 10 min, and subjected to SDS-PAGE on 4 to 15% Mini-PROTEAN TGX Precast Protein Gels (Bio-Rad, #4561085) or on 7.5% Mini-PROTEAN TGX Precast Protein Gels (Bio-Rad, #4561025). PageRuler Prestained Protein Ladder (Thermo Fisher Scientific, 26616) was used as a molecular weight marker. Separated proteins were transferred to polyvinylidene fluoride membranes (Merck, IPVH00010) and membranes were blocked in Tris-buffered saline with Tween 20 (TBST) + 5% nonfat dry milk (NFDM) for 1 h. Blots were incubated at 4 °C overnight with the primary antibody (RECK (D8C7) Rabbit mAb, Cell Signaling Technologies, #3433; anti-mouse GPR124 ECD (17); LRP6 (C47E12) Rabbit mAb, Cell Signaling Technologies, #3395; Phospho-LRP6 (Ser1490) Antibody, Cell Signaling Technologies, #2568; β-actin (AC-15) Mouse mAb, Merck, A1978; anti-WNT7A antibody (EPR23471-125), Abcam, ab274321; LRP5 (D80F2) Rabbit mAb, Cell Signaling Technologies, #5731; Dvl2 Antibody, Cell Signaling Technologies, #3216) diluted in TBST + 5% NFDM or 5% bovine serum albumin according to the manufacturer's instructions. Membranes were washed three times with TBST (10 min each), followed by incubation with the secondary antibody (anti-rabbit IgG-HRP, Dako/Agilent Technologies, P0448; anti-mouse IgG-HRP, Jackson ImmunoResearch Labs, 115-035-068) diluted 1:10000 in TBST + 5% NFDM. Blots were washed as before and incubated for 1 min with SuperSignal West Pico PLUS (Thermo Fisher Scientific, #34579) or SuperSignal West Femto PLUS Chemiluminescence Substrate (Thermo Fisher Scientific, #34095). Blots were imaged using the Fusion Solo X system (Vilber). For figures, dark field images (chemiluminescence) were inverted and superimposed with bright field images (protein ladder and membrane) using the Fusion Solo X software. For densitometry, only the inverted chemiluminescence images were used to reduce background. Band densities were quantified using ImageJ (version 1.53k). Specificity of the primary antibodies was validated using WT versus KO bEnd.3 cells.

### Generation of retroviral and lentiviral expression constructs

A retrovirus encoding the BirA biotin ligase was generated by subcloning secreted BirA-Flag (a gift from Gavin Wright, Addgene #64395) (42) into the retroviral expression vector pBABE-Puro-mTert (a gift from Marta Alvarez and Joseph Bidwell, Addgene #36413) (43), replacing the original insert (mTert). GP+E-86 retroviral packaging cells were seeded into a six-well plate (2 × 10^5^ cells per well) and transfected with 2 μg pBABE-Puro-BirA using the jetOPTIMUS transfection reagent (Polyplus, 101000025). Forty-eight hours post transfection, cells were selected for 14 days with 4 μg/ml puromycin (Thermo Fisher Scientific, A1113803). Stably transfected GP+E-86 cells were seeded into a T75 flask and incubated until confluent. Cells received fresh culture medium and were incubated for 48 h. The culture supernatant containing retroviral particles was harvested, passed through a 0.45 μm pore filter, and stored at −80 °C.

A lentivirus encoding a doxycycline-inducible biotin acceptor peptide (BAP)-RECK fusion protein was generated by inserting the BAP coding sequence into the human RECK complementary DNA immediately downstream of the signal peptide. A dsODN encoding the Kozak sequence, the human RECK signal peptide (aa 1-26), and the BAP tag (GLNDIFEAQKIEWHE) was synthesized (fragment 1, Thermo Fisher Scientific) and mature human RECK (aa 27-971) was amplified by PCR from p3xFLAG-CMV-9 hRECK (17) (fragment 2). The fragments were fused and subcloned into the lentiviral expression vector pCW-Cas9-BLAST (a gift from Mohan Babu, Addgene #83481) by Gibson assembly, replacing the original insert (Cas9). The resulting pCW-BAP-RECK-BLAST plasmid harbored the BAP-RECK fusion construct under control of the TRE tight promoter and rtTA advanced (Tet-On system). A total of 4 × 10^6^ HEK293T cells were seeded into a T25 flask and cotransfected with 1.4 μg pCW-BAP-RECK-BLAST, 1.4 μg psPAX2 (a gift from Didier Trono, Addgene #12260), and 0.9 μg pHCMV-EcoEnv (a gift from Miguel Sena-Esteves, Addgene #15802) (44) using the jetOPTIMUS transfection reagent (Polyplus, 101000025). Twenty-four hours after transfection, the cells received fresh culture medium and were incubated for 48 h. The culture supernatant containing lentiviral particles was harvested, passed through a 0.45 μm pore filter, and stored at −80 °C.

### Generation of bEnd.3 bRECK cells

bEnd.3 TCF-Luc *Reck*^*−/−*^ cells were seeded into a T75 flask at ∼70% confluency and incubated for 24 h with the BirA retroviral supernatant supplemented with 8 μg/ml polybrene (Merck, TR-1003). Transduction was repeated once, and cells were selected with 4 μg/ml puromycin (Thermo Fisher Scientific, A1113803) for 14 days. The resulting bEnd.3 TCF-Luc *Reck*^*−/−*^ BirA cells were seeded into a T25 flask at ∼70% confluency and incubated for 24 h with the BAP-RECK lentiviral supernatant supplemented with 8 μg/ml Polybrene (Merck, TR-1003). Transduction was repeated once, and cells were selected with 20 μg/ml blasticidin S (Thermo Fisher Scientific, R21001) for 7 days. To enrich for clones expressing bRECK, BAP-RECK expression was induced with 1 μg/ml doxycycline (Thermo Fisher Scientific, J60422.06), and cells were panned in a 10 cm Petri dish coated with neutravidin (Thermo Fisher Scientific, 31000). Panned cells were subjected to limiting dilution cloning. Several single-cell clones were tested for bRECK expression and function by western blotting, streptavidin shift, and WNT/β-catenin reporter assays. The clone with the highest WNT7 responsiveness was used for downstream experiments. bEnd.3 bRECK cells were continuously cultured in medium supplemented with 1 μg/ml doxycycline (Thermo Fisher Scientific, J60422.06) to prevent silencing of the TRE tight promoter (45). Unless stated otherwise, for experiments, doxycycline was withdrawn for 72 h, which reduced BAP-RECK protein expression to near endogenous RECK levels (basal expression from the TRE tight promoter).

To generate a *Gpr124*^*−/−*^ subline, bEnd.3 bRECK cells were cotransfected with the corresponding CRISPR construct and a GFP plasmid at a 1:1 ratio (see above). Single-cell clones exhibiting transient GFP expression were isolated by limiting dilution cloning. Isolated clones were tested for *Gpr124* knockout by western blotting and WNT/β-catenin reporter assays. One complete knockout clone was identified and used for downstream experiments (bEnd.3 bRECK *Gpr124*^*−/−*^ cells).

### Streptavidin pull-down

bEnd.3 bRECK, bEnd.3 bRECK *Gpr124*^*−/−*^, or bEnd.3 *Reck*^−/−^ BirA (negative control) cells were seeded into eight T175 flasks (3 × 10^6^ cells per flask) and incubated until confluent. Culture medium was replaced with doxycycline-free medium to reduce BAP-RECK expression to near endogenous RECK levels and medium was supplemented with 100 μM biotin (Merck, B4639-100MG). Cells were incubated until confluent (48 h). The monolayers were cocultured with nonadherent HEK293 WNT7A or parental control cells (2 × 10^7^ cells per flask) for 24 h. bEnd.3 *Reck*^−/−^ BirA negative control cells were only cocultured with parental HEK293 cells. Cells were washed once with ice-cold PBS, which completely removed the nonadherent HEK293 cells. bEnd.3 cells were incubated with the thiol-cleavable chemical crosslinker DSP (Thermo Fisher Scientific, #22586) at 0.1 mM in PBS at 4 °C for 2 h. Unreacted crosslinker was quenched with 20 mM Tris–HCl, pH 7.5 at 4 °C for 15 min, followed by washing the cells twice with ice-cold PBS. Cells were scraped into ice-cold RIPA buffer (25 mM Tris–HCl, pH 7.4; 150 mM NaCl; 1% Triton X-100; 0.5% sodium deoxycholate; and 0.1% SDS) supplemented with cOmplete ULTRA protease inhibitor (Merck, 5892970001) and PhosSTOP phosphatase inhibitor (Merck, 4906845001) cocktails and incubated on ice for 30 min, vortexing occasionally. Cell lysates were cleared by high-speed centrifugation. After taking small input samples, cell lysates (∼10 ml each) were supplemented with 100 μl Pierce Streptavidin Agarose (Thermo Fisher Scientific, #20347) and incubated under constant rotation at 4 °C overnight. Streptavidin beads were washed six times with 10 ml TBS + 2% SDS at room temperature. Crosslinked bRECK-associated proteins were eluted (eluate 1) by incubating the beads with 1 ml 2% SDS + 100 mM DTT at 50 °C for 30 min. Eluate 1 was separated from the beads using centrifugal filters. Bound bRECK was eluted (eluate 2) by incubating the beads with 100 μl reducing (100 mM DTT) Laemmli sample buffer at 95 °C for 10 min. Eluate 1 was subjected to mass spectrometry analysis. Inputs and both eluates were analyzed by western blotting.

### Mass spectrometry

Mass spectrometry was performed by the Mass Spectrometry Core Facility of the University of Würzburg. Briefly, protein samples were separated by SDS-PAGE, followed by Coomassie blue staining. Each lane was cut into 15 equal sections, proteins were digested in-gel with trypsin, and peptides were extracted. Resulting samples were analyzed using a nanoLC-coupled ESI-MS/MS system. MS/MS spectra were assigned to peptides and mapped to the mouse reference proteome (UniProtKB, taxon ID: 10090) using the MaxQuant software (version 1.6.2.2). Proteins were quantified by LFQ based on MS^1^ precursor ion intensities *via* the MaxLFQ algorithm. Proteins flagged as “Only identified by site”, “Reverse”, or “Potential contaminant”, proteins found in the negative control (LFQ intensity > 0), and proteins with a low total peptide count across all samples (<7) were excluded from downstream analyses to reduce background noise. After applying these filter criteria, a total of 134 RECK-associated proteins across all four conditions were identified. Proteins relevant to WNT/β-catenin signaling were identified by querying the GO term “canonical Wnt signaling pathway” (GO:0060070). Unbiased GO Molecular Function analysis for each condition was performed using the enrichGO function of the clusterProfiler R package (version 4.12.6) (46) in combination with the org.Mm.eg.db R package (GO source date: 2024-01-17).

### Clustering assays

#### HEK293 cells

HEK293 *RECK*^*−/−*^ cells were generated and transiently transfected with a WNT/β-catenin reporter (firefly luciferase), *Renilla* luciferase, WNT7B, RECK, and/or GPR124 for 24 h, as previously described (17). Cells were seeded into 96-well plates and incubated with purified recombinant GPR124 ECD-Fc (human GPR124 ECD [aa 34-755] fused to mouse IgG2a Fc [aa 237-469]) (17) or FLAG-GPR124 ECD (3xFLAG tag fused to mouse GPR124 ECD [aa 34-753]) (47) at 0, 1, 10, and 100 nM for 24 h. A dual-luciferase reporter assay (Promega, E1960) was performed according to the manufacturer's instructions. Firefly luciferase activity was normalized with *Renilla* luciferase activity and is shown relative to the activity in control cells.

#### bEnd.3 cells

Briefly, 1 × 10^4^ doxycycline-induced (1 μg/ml) bEnd.3 bRECK cells with or without *Gpr124* knockout were seeded per well into 96-well plates and incubated overnight until confluent. Cells were overlaid with 2 × 10^4^ HEK293 WNT7A or parental control cells and incubated for 12 h with either an anti-BAP tag antibody (GenScript, A00674) or a rabbit IgG isotype control (Invitrogen, 02-6102) at 0, 0.01, 0.1, 1, or 10 μg/ml. Luciferase reporter activity was determined as described above.

### Statistical analyses

For WNT reporter assays and western blot densitometry, an ordinary one- or two-way ANOVA (as specified in the figure legends) followed by Tukey’s multiple comparisons test was performed using GraphPad Prism (version 10.5.0). Only relevant comparisons as indicated in the figure legends are shown (ns, not significant; ∗*p* ≤ 0.05, ∗∗*p* ≤ 0.01, ∗∗∗*p* ≤ 0.001). Full statistical results are provided in Table S4. For GO molecular function term enrichment, a hypergeometric test with Benjamini-Hochberg correction was conducted using the enrichGO function of the clusterProfiler R package (version 4.12.6). For all analyses, adjusted *p*-values ≤ 0.05 were considered statistically significant.

## Data availability

All data supporting the findings of this study are included in the article and its supplementary information. Generated plasmids and cell lines are available from the corresponding author upon reasonable request (mario.vallon@uni-wuerzburg.de).

## Supporting information

This article contains supporting information.

## Conflict of interest

The authors declare that they have no conflicts of interest with the contents of this article.
