# Supplementary material for: WNT7A/B assemble a GPR124-RECK-LRP5/6 coreceptor complex to activate β-catenin signaling in brain endothelial cells
Source: J Biol Chem. 2025 Sep 4;301(10):110682. doi: 10.1016/j.jbc.2025.110682 (PMC12514574; doi:10.1016/j.jbc.2025.110682)
Supplement: Supplemental Figures [file mmc1.pdf]

Figure S1

A

Gpr124 KO

		DNA sequence (antisense strand)	Protein sequence
	WT ref.	CAGAGCACTTGCAACCGCGGATAGGGACCGGGCAGCCAGG	PGCPVPIRGCKCS
Clone 1	Allele 1	CAGAGCACTTGCAACCGCG-ATAGGGACCGGGCAGCCAGG	PGCPVPIAVASAL
	Allele 2	CAGAGCACTTGCAACCGCGG-TAGGGACCGGGCAGCCAGG	PGCPVPTAVASAL
Clone 2	Allele 1	CAGAGCACTTGCAACCGCG-ATAGGGACCGGGCAGCCAGG	PGCPVPIAVASAL
	Allele 2	CAGAGCACTTGCAACCGCGGGATAGGGACCGGGCAGCCAG	PGCPVPIPRLQVL
	Allele 3	CAGAGCACTTGC-------GATAGGGACCGGGCAGCCAGG	PGCPVPI--ASAL

B

Reck KO

		DNA sequence	Protein sequence
	WT ref.	GAGGTGACGGGGGGCCTGGCCCCGGGCAGCGCGGGTGCTG	EVTGGLAPGSAGA
Clone 1	Allele 1	GAGGTGACGGGGGGCCTG-CCCCGGGCAGCGCGGGTGCTG	EVTGGLPRAARVL
	Allele 2	GAGGTGACGGGGGGCCTGGCC--GGGCAGCGCGGGTGCTG	EVTGGLAGQRGC
Clone 2	Allele 1	GAGGTGACGGGGGGCCTGG-CCCGGGCAGCGCGGGTGCTG	EVTGGLARAARVL

C

Lrp5/6 KO – *Lrp5* target site

		DNA sequence	Protein sequence
	WT ref.	GCCCAATGGGCTGACCATCGACCTGGAGGAACAGAAGCTG	PNGLTIDLEEQKL
Clone 1	Allele 1	GCCCAATGGGCTGACCA---ACCTGGAGGAACAGAAGCTG	PNGLT-NLEEQKL
	Allele 2	GCCCAATGGGCTGA------ACCTGGAGGAACAGAAGCTG	PNGL--NLEEQKL
	Allele 3	GCCCAATGGGCTG-------ACCTGGAGGAACAGAAGCTG	PNGLT--WRNRS
	Allele 4	GCCCAATGGGCTGACCATC-ACCTGGAGGAACAGAAGCTG	PNGLTITWRNRS
Clone 2	Allele 1	GCCCAATGGGCTGACCACCG--------------------	PNGLTT-------
	Allele 2	GCCCAATGGGCTGACCATC----TGGAGGAACAGAAGCTG	PNGLTI-WRNRS

D

Lrp5/6 KO – *Lrp6* target site

		DNA sequence (antisense strand)	Protein sequence
	WT ref.	ATGGGATCTAACACGATAGCCCGGGGCTCCTCTAAGTCCT	DLEEPRAIVLDP
Clone 1	Allele 1	ATGGGATCTAACACGATAGCC-GGGGCTCCTCTAAGTCCT	DLEEPRLSC*
Clone 2	Allele 1	ATGGGATCTA----------CCGGGGCTCCTCTAAGTCCT	DLEEPR*
	Allele 2	ATGGGATCTAACACGATAGC--GGGGCTCCTCTAAGTCCT	DLEEPRYRVRSH

Figure S1 (continued)

E

Fzd1/2/4/5/7/8/9 KO – *Fzd1* target site

		DNA sequence (antisense strand)	Protein sequence
	WT ref.	GGGACAGGATCACCCACCAGATGGAGCTGGCCATGCTGAA	FSMASSIWWVILS
Clone 1	Allele 1	GGGACAGGATCACCCACC––––––––––GGCCATGCTGAA	FSMAGG*
Clone 1	Allele 2	GGGACAGGATCACCCACC–––TGGAGCTGGCCATGCTGAA	FSMASS–RWVILS
Clone 1	Allele 3	GGGACAGGATCACCCACC–GATGGAGCTGGCCATGCTGAA	FSMASSIGG*
Clone 1	Allele 4	GGGACAGGATCACCCAC–––––––––CTGGCCATGCTGAA	FSMA–––RWVILS
Clone 2	Allele 1	GGGACAGGATCACCCACC–GATGGAGCTGGCCATGCTGAA	FSMASSIGG*
Clone 2	Allele 2	GGGACAGGATCAC–––––AGATGGAGCTGGCCATGCTGAA	FSMASSI–CDPV
Clone 2	Allele 3	GGGACAGGATCACCCAC––GATGGAGCTGGCCATGCTGAA	FSMASSIVGDPV
Clone 2	Allele 4	GGGACAGGATCACCCACC–––––––––––––––––CTGAA	F–––––RVGDPV

F

Fzd1/2/4/5/7/8/9 KO – *Fzd2* target site

		DNA sequence	Protein sequence
	WT ref.	TTCTTCAGCATGGCCAGCTCCATCTGGTGGGTGATTCTGT	FFSMASSIWWVIL
Clone 1	Allele 1	TTCTTCAGCATGGCCAGCTCCAT––GGTGGGTGATTCTGT	FFSMASSMVGDS
Clone 1	Allele 2	TTCTTCAGCA–––––––––––––––––TGGGTGATTCTGT	FFSM––––––GDS
Clone 2	Allele 1	TTCTTCAGCATGGCCAGCTCCATCTTGGTGGGTGATTCTG	FFSMASSILVGDS
Clone 2	Allele 2	TTCTTCAGCATGGCCAGCTCCAT–––GTGGGTGATTCTGT	FFSMASS–MWVIL
Clone 2	Allele 3	TTCTTCAGCATGGCCAGCTCCATC––––GGGTGATTCTGT	FFSMASSIG*
Clone 2	Allele 4	TTCTTCAGCATGGCCAGCTCCA–––––TGGGTGATTCTGT	FFSMASS–MGDS

G

Fzd1/2/4/5/7/8/9 KO – *Fzd4* target site

		DNA sequence	Protein sequence
	WT ref.	TTTTGGAATGGCCAGCTCCATTTGGTGGGTTATTCTGACA	FGMASSIWWVILT
Clone 1	Allele 1	TTTTGGAATGGCCAGCTCC–––––GTGGGTTATTCTGACA	FGMASS–VGYSD
Clone 1	Allele 2	TTTTGGAATGGCCAGCTCCAT–––GTGGGTTATTCTGACA	FGMASS–MWVILT
Clone 1	Allele 3	TTTTGGAATGGCCA–––––––––GGTGGGTTATTCTGACA	FGMA–––RWVILT
Clone 1	Allele 4	TTTTGGAATGGCCAGCTCCAT––GGTGGGTTATTCTGACA	FGMASSMVGYSD
Clone 2	Allele 1	TTTTGGAATGGCCAGCTCCATT–GGTGGGTTATTCTGACA	FGMASSIGGLF*
Clone 2	Allele 2	TTTTGGAATGGCCAGCTC––––––––––––––––CTGACA	FGMASS*
Clone 2	Allele 3	TTTTGGAATGGCCAGCTCCA–––––TGGGTTATTCTGACA	FGMASS–MGYSD
Clone 2	Allele 4	TTTTGGAATGGCCAGCTCCA––––––––GTTATTCTGACA	FGMASS––SYSD

H

Fzd1/2/4/5/7/8/9 KO – *Fzd5* target site

		DNA sequence (antisense strand)	Protein sequence
	WT ref.	ACAGGATGACCCACCAGATGGAGCTGGCCATGCCAAAGAA	FFGMASSIWWVIL
Clone 1	Allele 1	ACAGGATGACCC––––––––––––––GCCATGCCAAAGAA	FFGM––––AGHP
Clone 1	Allele 2	ACAGGAT––––––––––––––––CTGGCCATGCCAAAGAA	FFGMA–––––RSC
Clone 1	Allele 3	ACAGGATGACCCACC––ATGGAGCTGGCCATGCCAAAGAA	FFGMASSMVGHP
Clone 1	Allele 4	ACAGGAT–––––––––––––GAGCTGGCCATGCCAAAGAA	FFGMASS––––SC
Clone 2	Allele 1	ACAGGATGACCCAC–––ATGGAGCTGGCCATGCCAAAGAA	FFGMASS–MWVIL
Clone 2	Allele 2	ACAGGATGACCCAC–AGATGGAGCTGGCCATGCCAAAGAA	FFGMASSICGSSC
Clone 2	Allele 3	ACAGGATGACCCACC––––GGAGCTGGCCATGCCAAAGAA	FFGMASS–GGSSC

Figure S1 (continued)

I

Fzd1/2/4/5/7/8/9 KO – *Fzd7* target site

		DNA sequence	Protein sequence
	WT ref.	CGGTATGGCCAGCTCCATCTGGTGGGTCATTCTGTCCCTC	GMASSIWWVILSL
Clone 1	Allele 1	CGGTATGGCCAGCTCCATC–GGTGGGTCATTCTGTCCCTC	GMASSIGGSFCP
	Allele 2	CGGTATGGCCAGCTCCATC–––TGGGTCATTCTGTCCCTC	GMASSI–WVILSL
	Allele 3	CGGTATGGCCAGCTCCATC––GTGGGTCATTCTGTCCCTC	GMASSIVGHSVP
Clone 2	Allele 1	CGGTATGGCCAGCTCCAT––GGTGGGTCATTCTGTCCCTC	GMASSMVGHSVP
	Allele 2	CGGTATGGCCAGCTCC––––––TGGGTCATTCTGTCCCTC	GMASS––WVILSL
	Allele 3	CGGTATGGCCAGCTCC––––GGTGGGTCATTCTGTCCCTC	GMASS–GGSFCP

J

Fzd1/2/4/5/7/8/9 KO – *Fzd8* target site

		DNA sequence	Protein sequence
	WT ref.	TGGCATGGCCAGCTCCATCTGGTGGGTAATCCTGTCGCTC	GMASSIWWVILSL
Clone 1	Allele 1	TGGCATGGCCAGCTCC–––––––––––––––––GTCGCTC	GMASS–––––VA
	Allele 2	TGGCATGGCCAGCTCCAT––GGTGGGTAATCCTGTCGCTC	GMASSMVGNPVA
	Allele 3	TGGCATGGCCAG––––––––GGTGGGTAATCCTGTCGCTC	GMA––RVGNPVA
Clone 2	Allele 1	TGGCATGGCCAGCTCCATC–GGTGGGTAATCCTGTCGCTC	GMASSIGG*
	Allele 2	TGGCATGGCCAG––––––––GGTGGGTAATCCTGTCGCTC	GMA––RVGNPVA
	Allele 3	TGGCATGGCCAGCTCC––––GGTGGGTAATCCTGTCGCTC	GMASSGG*

K

Fzd1/2/4/5/7/8/9 KO – *Fzd9* target site

		DNA sequence (antisense strand)	Protein sequence
	WT ref.	GCGCGAAGTCCTTATCGCGCCGAGACCAGAACACCTCGAC	VEVFWSRRDKDFA
Clone 1	Allele 1	GCGCGAAGTCCTTAT––CGCCGAGACCAGAACACCTCGAC	VEVFWSRR*
	Allele 2	GCGCGAAGTCCTTAT–GCGCCGAGACCAGAACACCTCGAC	VEVFWSRRIRTSR
	Allele 3	GCGCGAAGTCC––––CGCGCCGAGACCAGAACACCTCGAC	VEVFWSRR–GTSR
	Allele 4	GCGC–––––––––––CGCGCCGAGACCAGAACACCTCGAC	VEVFWSRR––––G
Clone 2	Allele 1	GCGCGAAGTCCTTAT––CGCCGAGACCAGAACACCTCGAC	VEVFWSRR*
	Allele 2	GCGCGAAGTC–––––––––––––––CCAGAACACCTCGAC	VEVFW–––––DFA
	Allele 3	GCGCGAAGTCCTTAT–GCGCCGAGACCAGAACACCTCGAC	VEVFWSRRIRTSR
	Allele 4	GCGCGAAGTCCTTA–––CGCCGAGACCAGAACACCTCGAC	VEVFWSRR–KDFA

Figure S1 (continued)

L

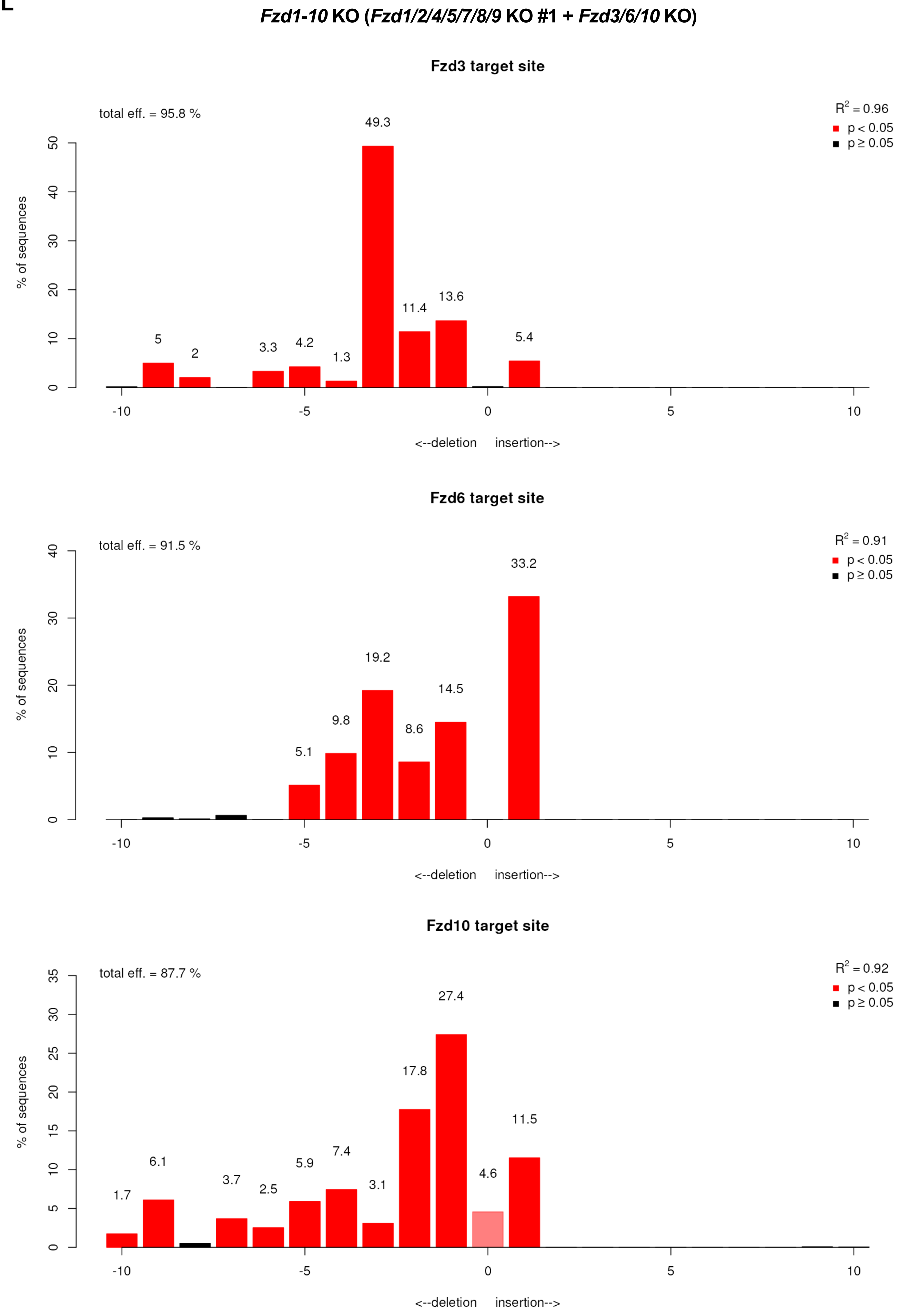

Figure S1 (continued)

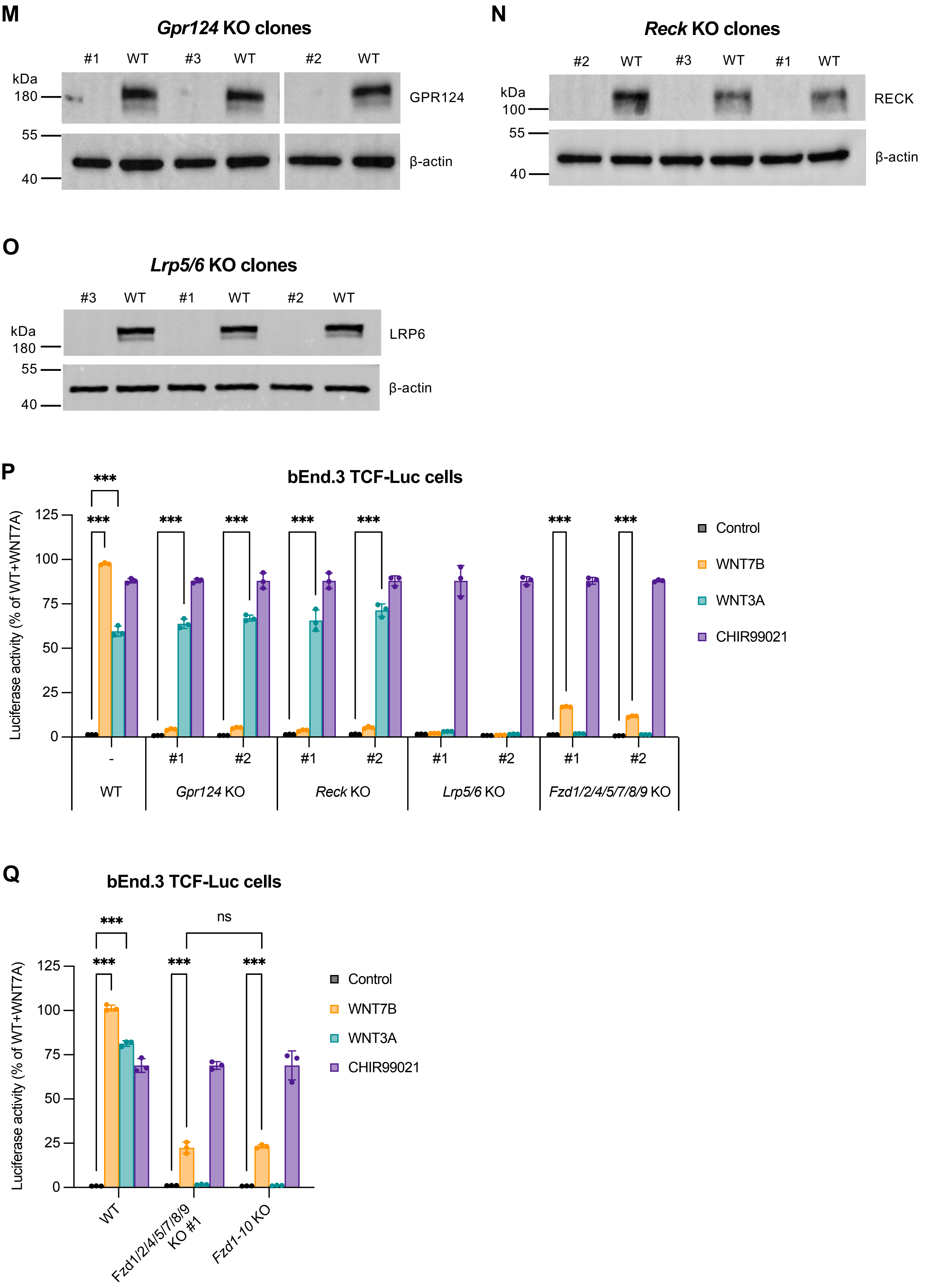

Figure S1. Characterization of WNT7 receptor knockouts in bEnd.3 TCF-Luc cells. Related to Figures 1 and 2.

(A-K) Sanger sequencing (left) and protein translation (right) of CRISPR target sites in KO clones of tetraploid bEnd.3 TCF-Luc cells. Mixed sequencing chromatograms were deconvoluted using the CRISP-ID software or DECODR online tool. Left: Blue and red lines mark the gRNA sequence and PAM site, respectively; dashes represent deletions, green shading indicates insertions. Right: Red amino acids represent protein sequence mismatches, and asterisks denote premature stop codons.

(L) TIDE analysis of CRISPR target sites in *Fzd1-10* KO bEnd.3 TCF-Luc cells. Cells were derived from *Fzd1/2/4/5/7/8/9* KO clone 1 with additional KO of *Fzd3, Fzd6,* and *Fzd10* (clone pool). Bars represent allelic indel frequencies (%).

(M-O) Western blot analysis of indicated KO clones of bEnd.3 TCF-Luc cells to confirm KO induction on the protein level.

(P-Q) WNT/β-catenin reporter gene assays using bEnd.3 TCF-Luc cells with indicated single or multiplex KOs. Numbers denote independent KO clones. *Fzd1-10* KO cells were derived from *Fzd1/2/4/5/7/8/9* KO clone #1 with additional KO of *Fzd3, Fzd6,* and *Fzd10* (clone pool). WT or KO cells were co-cultured for 24 h with non-adherent parental HEK293 cells (control) or HEK293 cells expressing WNT7B or WNT3A. Treatment with the GSK3 inhibitor CHIR99021 (10 μM) served as positive control. Bars represent luciferase activity normalized to CHIR99021 treament and expressed as % of WNT7A-stimulated WT cells (mean ± SD, n = 3, biological replicates). A two-way ANOVA with Tukey's multiple comparisons test was used for statistical analysis. Only significant differences between control and WNT7B or WNT3A stimulation within WT cells or KO clones are depicted. Full statistical results are provided in Table S4. ***p ≤ 0.001.

ref., reference; gRNA, guide RNA; PAM, protospacer adjacent motif; SD, standard deviation; CRISPR, clustered regularly interspaced short palindromic repeats; DECODR, deconvolution of complex DNA repair; TIDE, tracking of indels by decomposition.

Figure S2

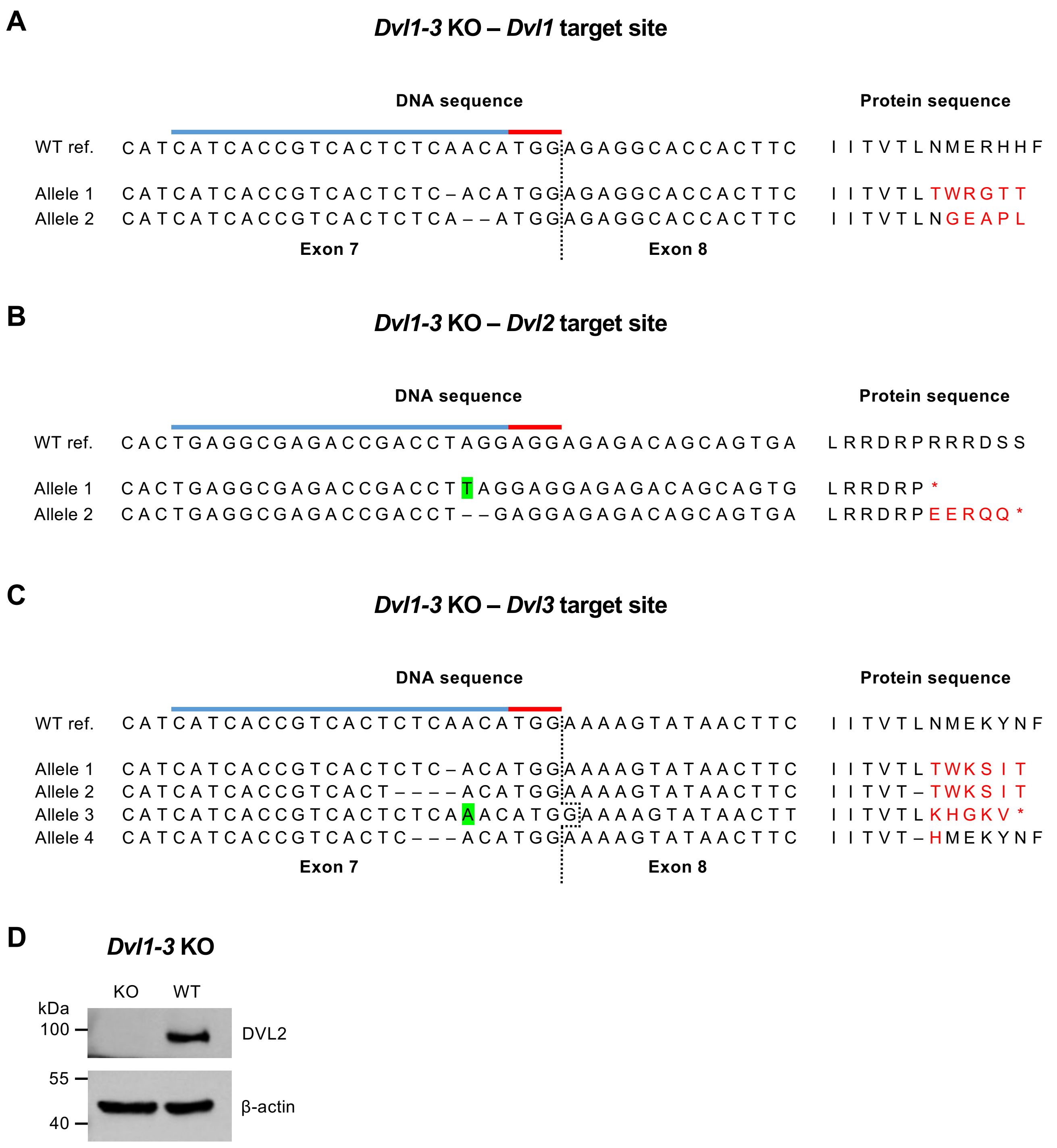

Figure S2. Characterization of *Dvl1-3* KO in bEnd.3 TCF-Luc cells. Related to Figure 3.
(A-C) Sanger sequencing (left) and protein translation (right) of indicated CRISPR target sites in a monoclonal *Dvl1-3* KO subline of bEnd.3 TCF-Luc cells. Mixed sequencing chromatograms were deconvoluted using the DECODR online tool. Left: Blue and red lines mark the gRNA sequence and PAM site, respectively; dashes represent deletions, green shading indicates insertions. In (A) and (C) exons 7 and 8 were spliced *in silico* to enable protein tranlation. Right: Red amino acids represent protein sequence mismatches, and asterisks denote premature stop codons.
(D) Western blot analysis of bEnd.3 TCF-Luc cells with *Dvl1-3* KO to confirm KO induction on the protein level.

ref., reference; gRNA, guide RNA; PAM, protospacer adjacent motif; SD, standard deviation; CRISPR, clustered regularly interspaced short palindromic repeats; DECODR, deconvolution of complex DNA repair.

Figure S3

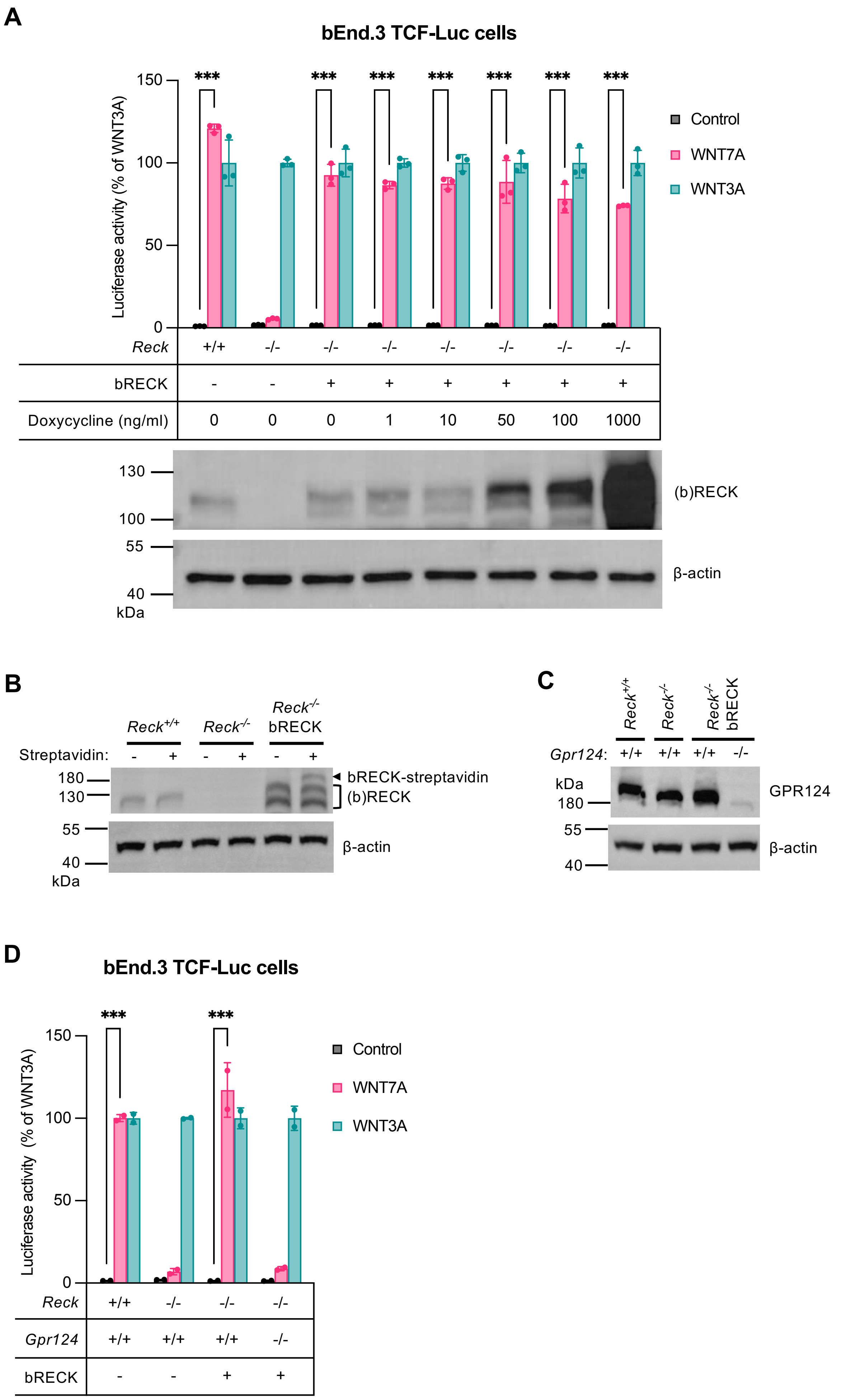
A
bEnd.3 TCF-Luc cells
Luciferase activity (% of WNT3A)
150
100
50
0
***
Control
WNT7A
WNT3A
Reck
+/+
-/-
bRECK
-
+
Doxycycline (ng/ml)
0
1
10
50
100
1000
130
100
55
40
kDa
(b)RECK
β-actin
B
Reck+/+
Reck-/-
Reck-/- bRECK
Streptavidin:
180
130
55
40
kDa
bRECK-streptavidin
(b)RECK
β-actin
C
Reck+/+
Reck-/-
Reck-/- bRECK
Gpr124:
+/+
-/-
kDa
180
55
40
GPR124
β-actin
D
bEnd.3 TCF-Luc cells
Luciferase activity (% of WNT3A)
150
100
50
0
***
Control
WNT7A
WNT3A
Reck
Gpr124
bRECK

Figure S3 (continued)

E

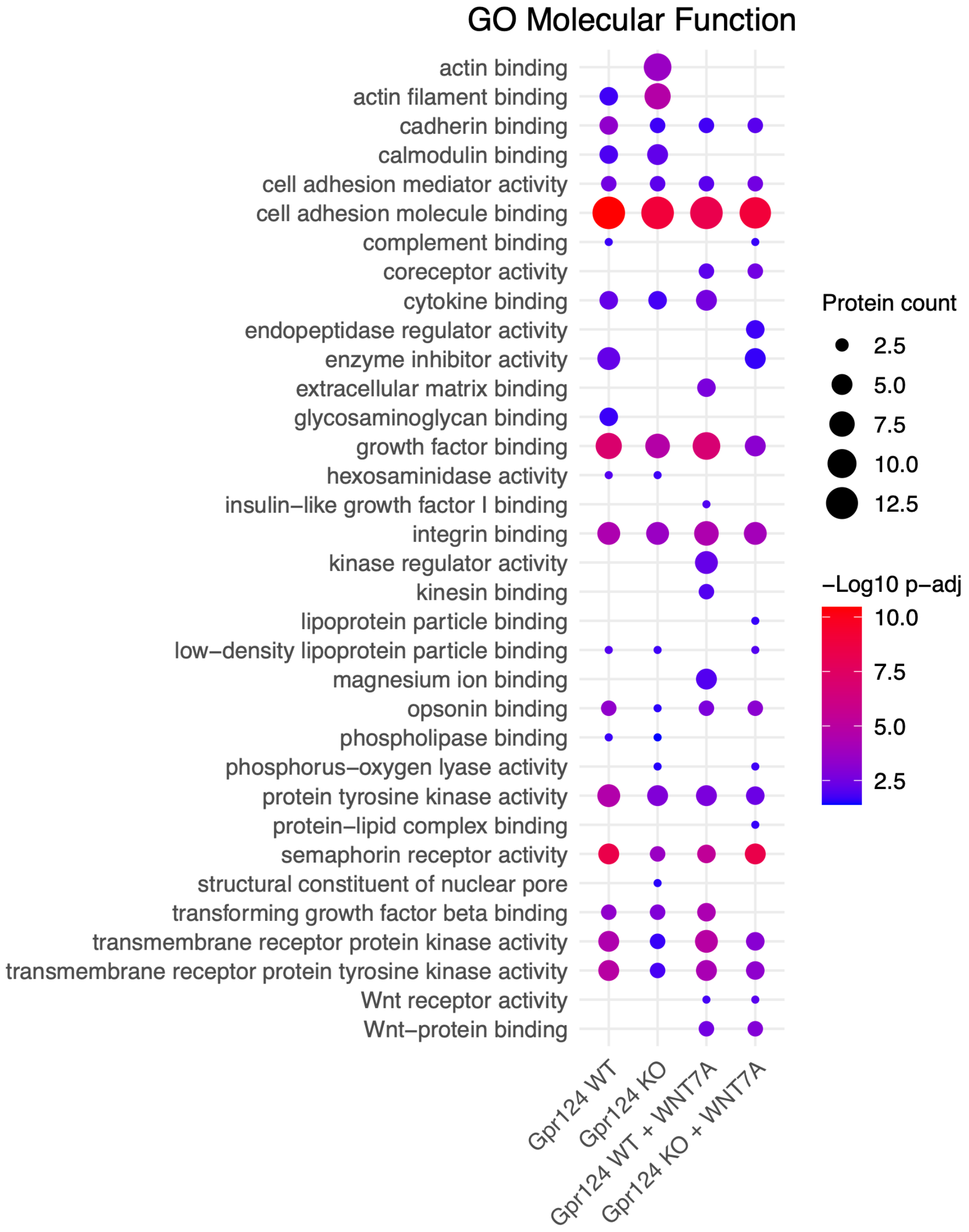

Figure S3. Characterization of bEnd.3 bRECK cells and GO analysis of bRECK-associated proteins. Related to Figure 4.

(A) WNT/β-catenin reporter gene assay (top) and Western blot analysis (bottom) using the indicated bEnd.3 TCF-Luc sublines. bRECK expression was induced by doxycycline (Tet-On system) at the indicated concentrations for 72 h. Cells were co-cultured for 24 h with non-adherent parental HEK293 cells (control) or HEK293 cells expressing WNT7A or WNT3A. Bars represent luciferase activity as % of WNT3A stimulation (mean ± SD, n = 3, biological replicates). A two-way ANOVA with Tukey's multiple comparisons test was used for statistical analysis. Only significant differences between control and WNT7A stimulation within each condition are shown. Full statistical results are provided in Table S4. ***p ≤ 0.001. For Western blotting, both endogenous RECK and bRECK were visualized using an anti-RECK antibody.

(B) Streptavidin-shift Western blot analysis of bEnd.3 bRECK cells. Where indicated (+), denatured protein samples (20 µg) were supplemented with streptavidin (1 µg) prior to SDS-PAGE. Endogenous RECK, bRECK, and the bRECK-streptavidin complex were visualized using an anti-RECK antibody.

(C) Western blot analysis of the indicated bEnd.3 TCF-Luc sublines.

(D) WNT/β-catenin reporter gene assay using the indicated bEnd.3 TCF-Luc sublines co-cultured for 24 h with non-adherent parental HEK293 cells (control) or HEK293 cells expressing WNT7A or WNT3A. Bars represent luciferase activity as % of WNT3A stimulation (mean ± SD, n = 2, biological replicates). A two-way ANOVA with Tukey's multiple comparisons test was used for statistical analysis. Only significant differences between control and WNT7A stimulation within each condition are shown. Full statistical results are provided in Table S4. ***p ≤ 0.001.

(E) GO Molecular Function analysis of bRECK and bRECK-associated proteins identified in bEnd.3 bRECK cells under the specified conditions. Dot size represents the number of proteins overlapping with each GO term. A hypergeometric test with Benjamini-Hochberg correction was used for statistical analysis. Color indicates the -log10 of the adjusted p-value (red: high significance, blue: low significance). Full results are provided in Table S3.

BAP, biotin acceptor peptide; bRECK, biotinylated BAP-RECK; SD, standard deviation; SA, streptavidin; GO, gene ontology; p-adj, adjusted p-value.

Figure S4

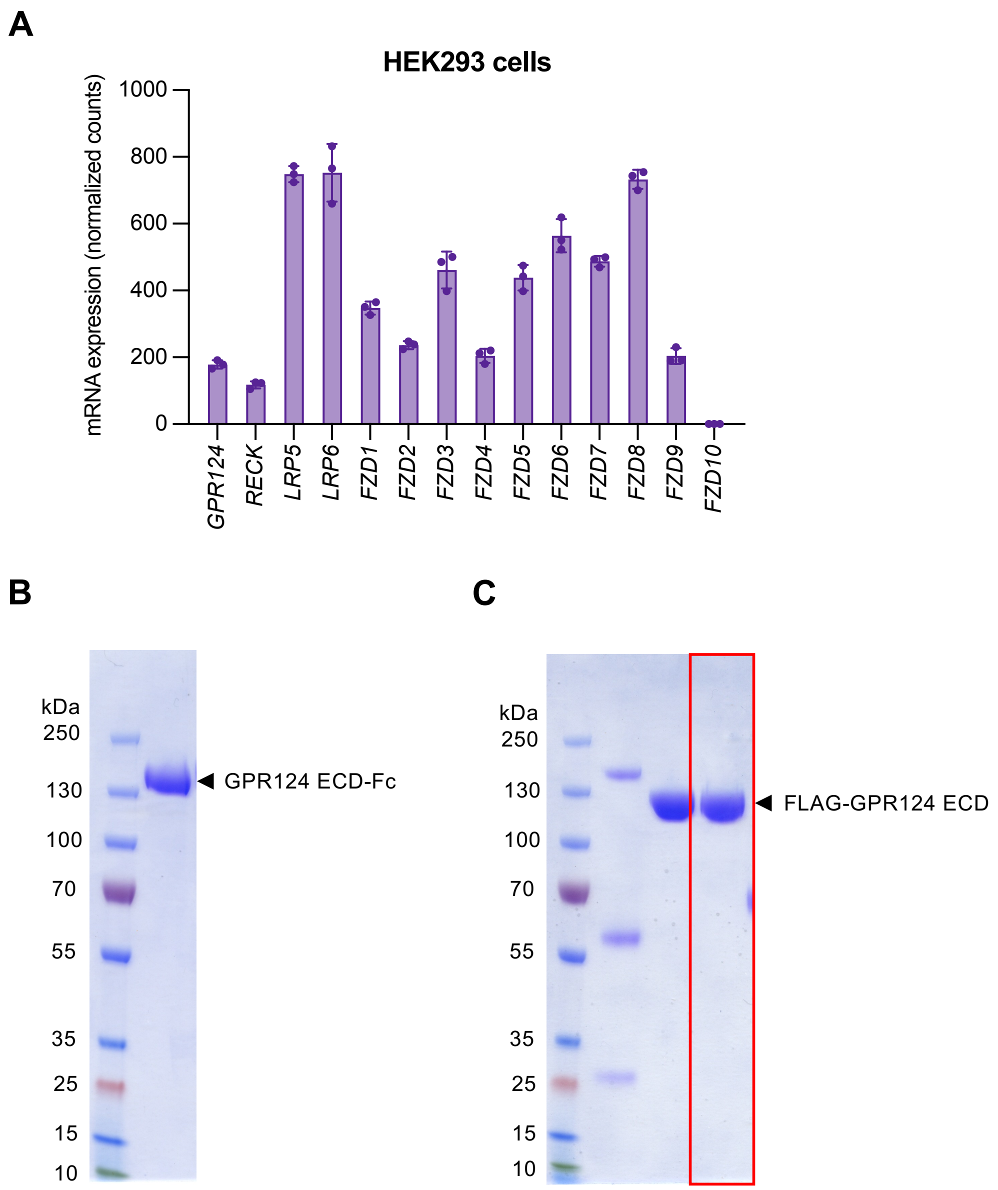

Figure S4. mRNA expression of WNT7 receptors in HEK293 cells and purification of recombinant GPR124 ECD proteins. Related to Figure 5.

(A) RNA-seq analysis of WNT7 receptors in HEK293 cells (GEO #GSE249290, HEK293WT confluent). Bars show mean normalized counts ± SD (n = 3, biological replicates).

(B-C) SDS-PAGE followed by Coomassie staining of **(B)** purified recombinant GPR124 ECD-Fc (human GPR124 ECD fused to mouse IgG2a Fc) and **(C)** purified recombinant FLAG-GPR124 ECD (3xFLAG tag fused to mouse GPR124 ECD). 3 µg protein per lane were loaded. The FLAG-GPR124 ECD lane is marked by a red box.

GEO, Gene Expression Omnibus; ECD, ectodomain; Fc, fragment crystallizable.

Figure S5

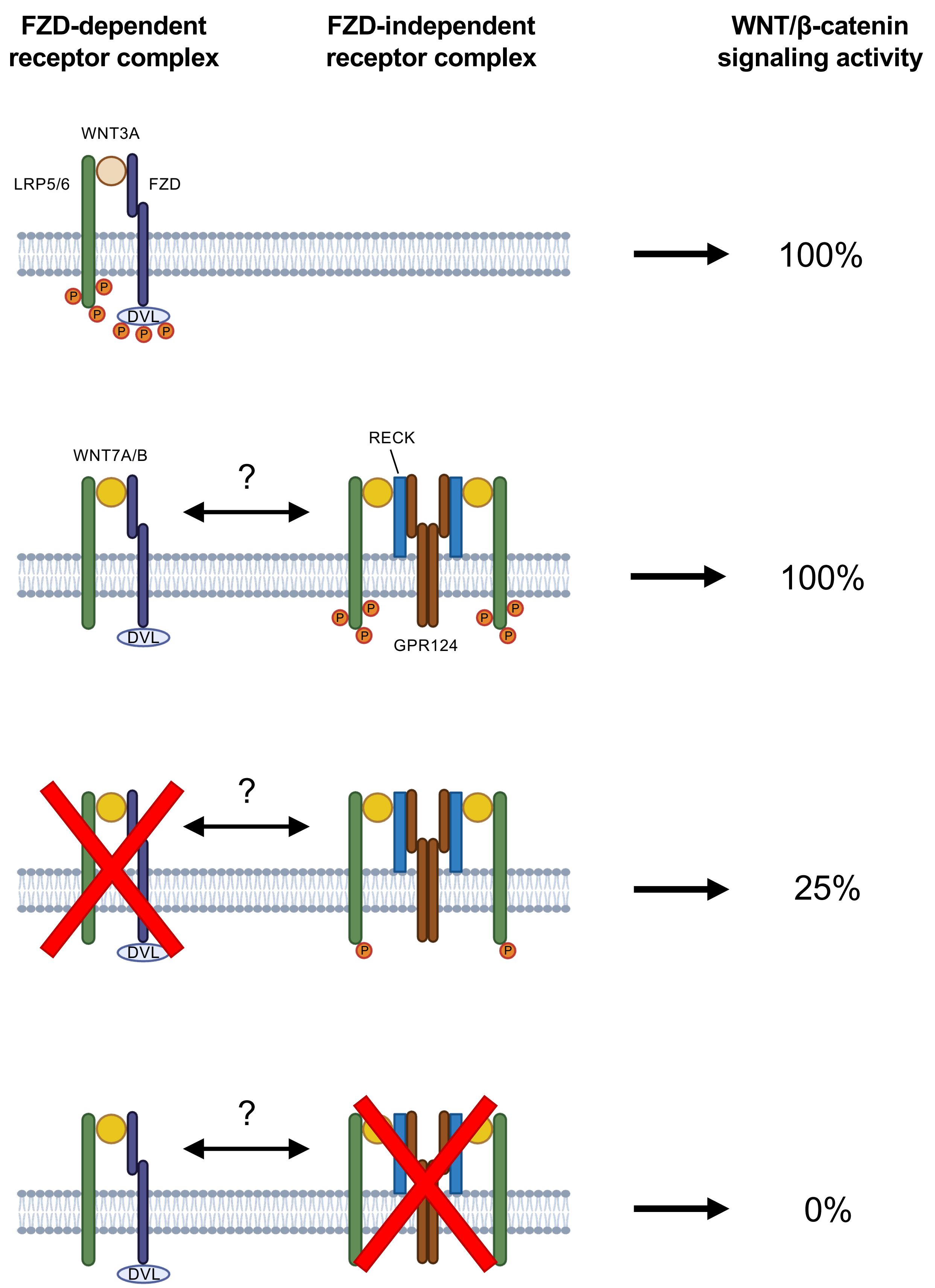

Figure S5. Graphical summary of the findings of this study. Dimerization of GPR124 is hypothetical. Created in BioRender. Heiden, R. (2025) https://BioRender.com/sznd0jc
