# Supplementary material for: WNT7A/B assemble a GPR124-RECK-LRP5/6 coreceptor complex to activate β-catenin signaling in brain endothelial cells
Source: J Biol Chem. 2025 Sep 4;301(10):110682. doi: 10.1016/j.jbc.2025.110682 (PMC12514574; doi:10.1016/j.jbc.2025.110682)
Supplement: Table S1 [file mmc2.pdf]

**Table S1. CRISPR/Cas9 gRNAs used for gene knockouts in bEnd.3 TCF-Luc cells. Related to Figures 1 and 2.** All gRNAs target the coding sequence of the corresponding gene(s). The *Fzd1/2/4/5/7/8* and *Dvl1/3* gRNAs target highly conserved regions across the respective paralogs. CRISPR, clustered regularly interspaced short palindromic repeats; gRNA, guide RNA; PAM, protospacer adjacent motif.

| **Target gene(s)** | **gRNA sequence** | **PAM** |
|---|---|---|
| *Gpr124* | AGCACTTGCAACCGCGGATA | GGG |
| *Reck* | GTGACGGGGGGCCTGGCCCC | GGG |
| *Lrp5* | CAATGGGCTGACCATCGACC | TGG |
| *Lrp6* | GGATCTAACACGATAGCCCG | GGG |
| *Fzd1/2/4/5/7/8* | CATGGCCAGCTCCATCTGGT | GGG |
| *Fzd3* | TTAATGCATCAACGTCGTAG | AGG |
| *Fzd6* | ATTCTTCTGCCCCTCGTAAG | AGG |
| *Fzd9* | GTTCTGGTCTCGGCGCGATA | AGG |
| *Fzd10* | GCACGAGTTCGCGCCGCTCG | TGG |
| *Dvl1/3* | CATCACCGTCACTCTCAACA | TGG |
| *Dvl2* | TGAGGCGAGACCGACCTAGG | AGG |
