# Supplementary material for: WNT7A/B assemble a GPR124-RECK-LRP5/6 coreceptor complex to activate β-catenin signaling in brain endothelial cells
Source: J Biol Chem. 2025 Sep 4;301(10):110682. doi: 10.1016/j.jbc.2025.110682 (PMC12514574; doi:10.1016/j.jbc.2025.110682)
Supplement: Table S2 [file mmc3.pdf]

Table S2. PCR and sequencing primers used to analyze CRISPR target sites in KO sublines of bEnd.3 TCF-Luc cells. Related to Figures 1 and 2. CRISPR, clustered regularly interspaced short palindromic repeats.

Target gene	Primer type	Primer sequence	Sequencing primer
Gpr124	Forward	GCTGCTGCCTTGTCTTCTGC	
	Reverse	GTCCCACTAATTGACCATACCC	X
Reck	Forward	CCAATCACCAGAGAGCCAAG	X
	Reverse	GCACCTCCCTGTTTTCTGAC	
Lrp5	Forward	TATCCCGTCTCCTCACTAGG	X
	Reverse	GCTCTGTTCCCAAAGTCATCTA	
Lrp6	Forward	ATCCGTCGCTCCTTCATAGA	
	Reverse	GACTCCAAGAGTTCATGTCACC	X
Fzd1	Forward	CTACATCGCTGGCTTTCTGTT	
	Reverse	CAGGAAAGAAGTGCCAATGAAC	X
Fzd2	Forward	CTTCACGGTCACCACCTATTT	X
	Reverse	CGATGAACAGGTATACGAAGAG	
Fzd3	Forward	GGCCTGTACCATGCTCTTTAT	X
	Reverse	TCTCTGTATTCCCTCACACTCT	
Fzd4	Forward	ATATTGTTCGGCTGACTGTAGG	X
	Reverse	CAGCGTTCCAATCACCAAATAC	
Fzd5	Forward	TCTGTGCTGTGCTTCATCTC	
	Reverse	GACAAAGCCTCGTAGTGAGT	X
Fzd6	Forward	GTGGATATGTCTCTTGCTAGG	X
	Reverse	CTAACCGTCCAACTGTAGACAA	
Fzd7	Forward	CTGTCGGGTTGCTACTTCAT	X
	Reverse	GGATGCGAAAGAGAGACA	
Fzd8	Forward	GAGCAGCATGTTCGCTATG	X
	Reverse	AAAGCCGCGTAGGTTGTCA	
Fzd9	Forward	TCATGGAGCAATTCAATTTCGG	
	Reverse	GAGTAGACATTGTAGCACATAG	X
Fzd10	Forward	CAGGTGATGATAGGCTCGTG	X
	Reverse	CTGAATTTGAACTGCTCCATGAT	
Dvl1	Forward	GTACGTGCTGGAGATTTGTAG	X
	Reverse	GCTCAATGCGGCCATCAG	
Dvl2	Forward	GTTGGAAACTCTCTCTACCTG	X
	Reverse	TGTCATCCTCATCCGAGTCT	
Dvl3	Forward	GAGGTTAGACTGTGAGTCCTTC	X
	Reverse	CCCAGAGCCTTGTACTATACTG	
